# Aptamer-based assay for high-throughput substrate profiling of RNA decapping enzymes

**DOI:** 10.1093/nar/gkae919

**Published:** 2024-10-24

**Authors:** Katarzyna Grab, Mateusz Fido, Tomasz Spiewla, Marcin Warminski, Jacek Jemielity, Joanna Kowalska

**Affiliations:** Division of Biophysics, Institute of Experimental Physics, Faculty of Physics, University of Warsaw, Pasteura 5, 02-093, Warsaw, Poland; Doctoral School of Exact and Natural Sciences, University of Warsaw, Żwirki i Wigury 93, 02-089, Warsaw, Poland; Division of Biophysics, Institute of Experimental Physics, Faculty of Physics, University of Warsaw, Pasteura 5, 02-093, Warsaw, Poland; Division of Biophysics, Institute of Experimental Physics, Faculty of Physics, University of Warsaw, Pasteura 5, 02-093, Warsaw, Poland; Doctoral School of Exact and Natural Sciences, University of Warsaw, Żwirki i Wigury 93, 02-089, Warsaw, Poland; Division of Biophysics, Institute of Experimental Physics, Faculty of Physics, University of Warsaw, Pasteura 5, 02-093, Warsaw, Poland; Centre of New Technologies, University of Warsaw, Banacha 2c, 02-097, Warsaw, Poland; Division of Biophysics, Institute of Experimental Physics, Faculty of Physics, University of Warsaw, Pasteura 5, 02-093, Warsaw, Poland

## Abstract

Recent years have led to the identification of a number of enzymes responsible for RNA decapping. This has provided a basis for further research to identify their role, dependency and substrate specificity. However, the multiplicity of these enzymes and the complexity of their functions require advanced tools to study them. Here, we report a high-throughput fluorescence intensity assay based on RNA aptamers designed as substrates for decapping enzymes. Using a library of differently capped RNA probes we generated a decapping susceptibility heat map, which confirms previously reported substrate specificities of seven tested hydrolases and uncovers novel. We have also demonstrated the utility of our assay for evaluating inhibitors of viral decapping enzymes and performed kinetic studies of the decapping process. The assay may accelerate the characterization of new decapping enzymes, enable high-throughput screening of inhibitors and facilitate the development of molecular tools for a better understanding of RNA degradation pathways.

## Graphical Abstract

**Graphical Abstract:**
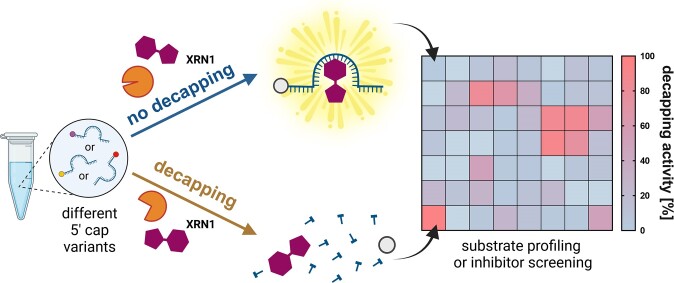

## Introduction

**Figure 1.**
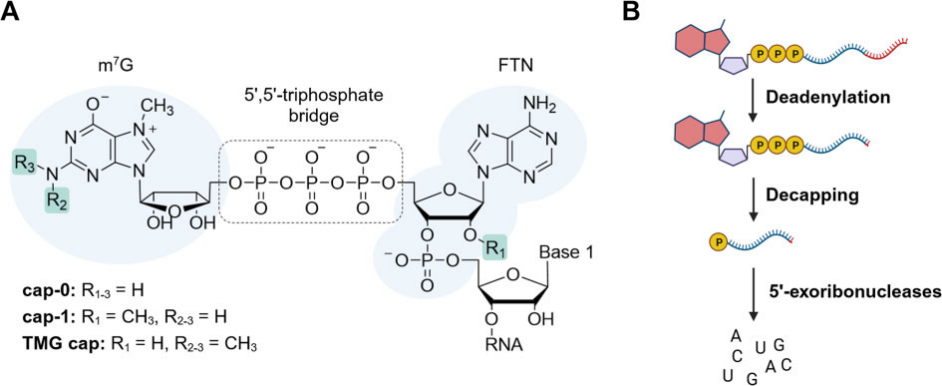
(**A**) Structure of the canonical cap at the 5′ end of mRNA. The figure highlights key structural elements and schematically depicts the mRNA 5′-to-3′ degradation pathway. (**B**) Schematic representation of mRNA decay in the 5′-to-3′ direction.

The presence of a cap structure at the 5′ end and the poly(A) tail at the 3′ end significantly increases the cellular stability of eukaryotic messenger RNAs (1). Because of these structural features, mRNA degradation involves multiple steps catalyzed by several enzymes, making them important players in the regulation of gene expression. The 5′ cap is responsible not only for protecting mRNA 5′ end from premature degradation (2) but also for cytoplasmic transport and translation initiation (3). It is therefore a determinant of which transcripts will be translated into proteins and which will be rapidly degraded by exonucleases. The canonical 5′ cap structure is formed by a 7-methylguanosine (m^7^G) linked to the first transcribed nucleotide (FTN) of the RNA sequence by a 5′,5′-triphosphate bridge (Figure 1A). In higher eukaryotes, the nascent cap-0 (m^7^GpppN) structure is additionally methylated at the ribose of the FTN to form a cap-1 (m^7^GpppN_m_), which is critical for differentiation between ‘self’ and ‘non-self’ RNAs, e.g. during viral infection (4). Another type of cap structure, a hypermethylated 2,2,7-trimethylguanosine (TMG) cap, is a hallmark of small nuclear RNAs (snRNAs) (5–8), but has also been found in several mammalian selenoprotein mRNAs bound to actively translating ribosomes and in some viral RNAs (9,7).

**Figure 2.**
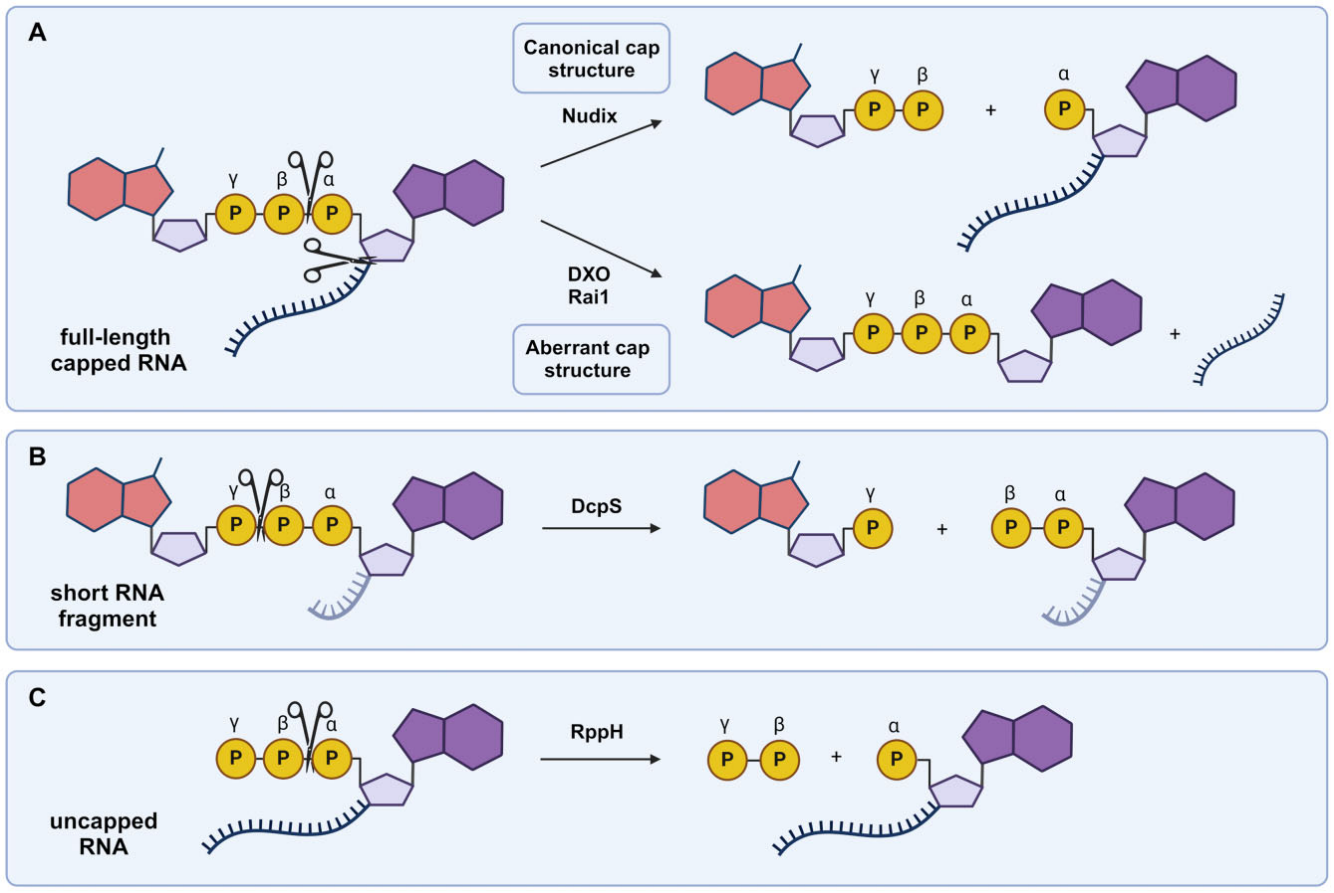
Different RNA cleavage sites by decapping enzymes depending on the nature of the substrate. (**A**) Full-length RNA containing canonical cap structure or a non-canonical/aberrant cap. (**B**) Short (<15 nt) RNA 5′ terminal fragment. (**C**) Uncapped RNA.

mRNA decay usually begins with the shortening of the poly(A) tail, which is a signal for the assembly of a decapping mRNP (messenger ribonucleoprotein) (10–12). The key step in the 5′-to-3′ degradation pathway is the hydrolysis of the 5′ cap, catalyzed by RNA-binding, hydrolytic proteins called ‘decapping enzymes’ (Figure 1B) (13). The best-characterized family of these proteins is the Nudix hydrolases, with its most notable member, Dcp2 (14). The enzyme hydrolyzes the triphosphate bridge within the cap structure (Figure 2A), releasing 7-methylguanosine diphosphate (m^7^GDP) and 5′-monophosphorylated RNA, which is then digested by 5′-exoribonucleases, such as Xrn1 (14). The Dcp2, in complexes with numerous binding partners that act as decapping enhancers (e.g. Dcp1, PNRC2, Edc3, etc.), is considered to be the major cellular decapping enzyme in eukaryotes, involved in bulk mRNA degradation (15–17). However, there are other Nudix enzymes (22 members have been found in the human genome (18)) that exhibit RNA decapping activity, at least *in vitro* (19), but their substrate specificity has not been fully characterized. Some eukaryotic viruses encode their own decapping enzymes, such as the D9 and D10 enzymes of the vaccinia virus, which also belong to the Nudix family (20). Nudix hydrolases have also been identified in bacteria (20,21): RppH shows activity toward triphosphate (Figure 2C) and cap-0 RNAs, and NudC preferentially cleaves NAD-capped RNAs (18,21).

Another group of enzymes with decapping activity is the decapping and exoribonuclease protein (DXO) family, which appears to be involved in mRNA quality control by degrading incompletely methylated, i.e. aberrant, cap structures, to release, e.g. GpppG from GpppG-RNAs (Figure 2A) (22). These enzymes have been shown to possess multiple activities, including decapping, 5′→3′ exoribonucleolytic and deNADding activity (18). Recently, mammalian DXO and its yeast homolog Rai1 have been shown to remove some other non-canonical cap structures, such as FAD and dephosphocoenzyme A (23). In each case, the resulting product is an RNA 5′-monophosphate that is exposed to further degradation by exonucleases.

Decapping enzymes are also required for the hydrolysis of cap residues released during 3′→5′ mRNA degradation. The prime example of such a scavenger enzyme is DcpS, a member of the histidine triad family. DcpS recognizes and hydrolyzes dinucleotide cap structures or short-capped RNA fragments, but it is not catalytically active toward longer RNAs (Figure 2B) (24).

**Figure 3.**
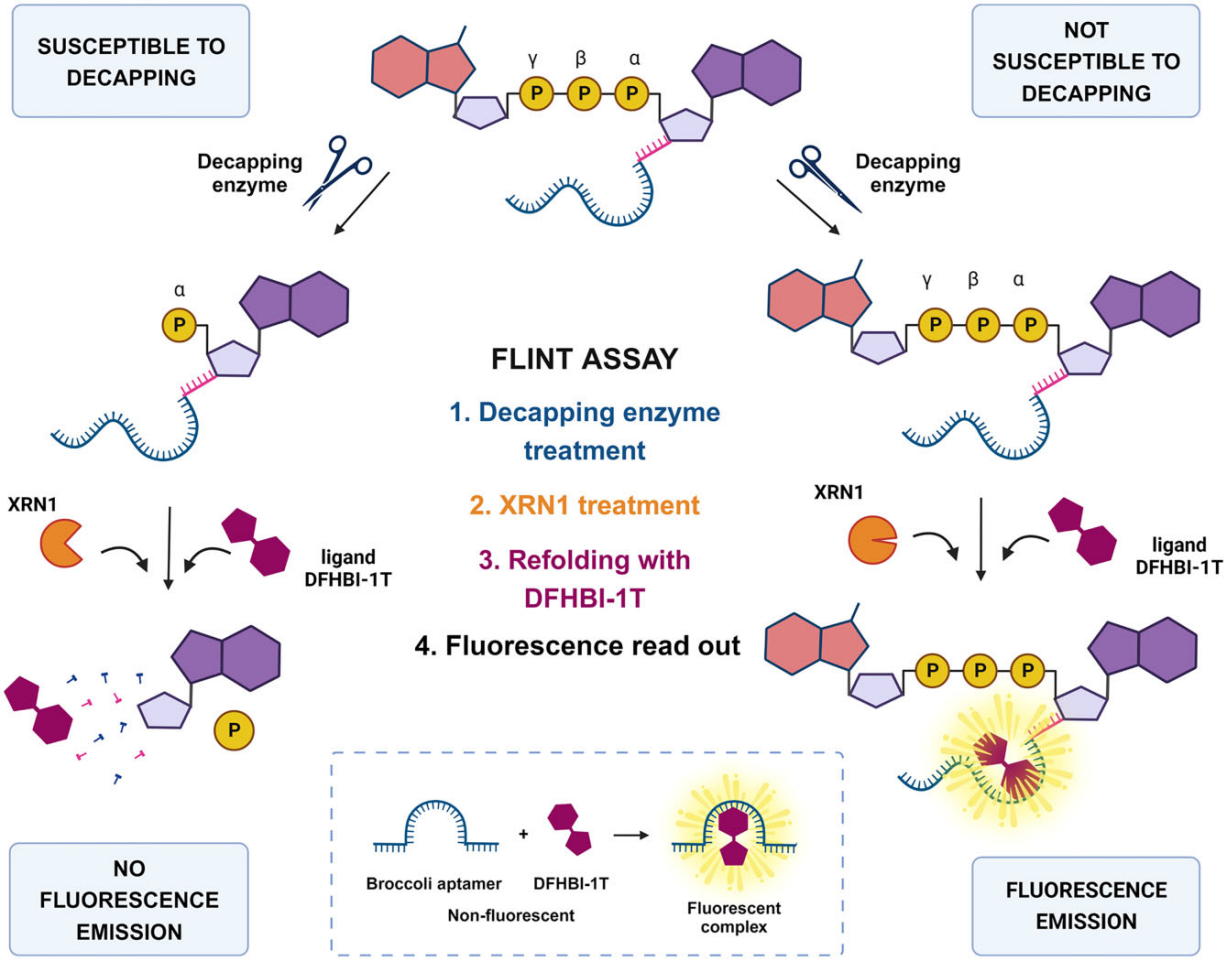
The FLINT assay for testing the decapping activity of enzymes. The diagram shows two pathways of possible interaction between aptamer probe capped with different cap analogs and decapping enzyme. If no activity occurs, the capped RNA remains intact (right pathway). If the protein removes the cap structure, RNA becomes susceptible to exonucleolytic cleavage from the 5′ end (left pathway). In either case, we incubate the sample with Xrn1, refold it, and incubate it with DFHBI-1T thereafter. Finally, we perform a fluorescence readout. If RNA remains intact, we observe high emission levels. Otherwise, we see a lowering in FLINT proportional to the decapping enzyme's activity.

Many decapping enzymes are important regulators of gene expression and dysregulation of their activity has been implicated in several human diseases, including spinal muscular atrophy (25), mental disability (26,27), Lafora disease (28) and cancer (29–32). Despite the important biological functions of the decapping enzymes, which make some of them promising therapeutic targets, there are still many unanswered questions about this diverse group of enzymes (33). Recent reports on the potential biological roles of non-canonical cap structures (34) as well as the variety of canonical cap variants (35), create the need to directly compare the activity of different decapping enzymes against differently capped RNAs. Such a back-to-back analysis would greatly benefit the field of RNA biology, but currently available tools are not suitable for such large-scale studies. Most of the decapping assays described in the literature to date are based on very laborious and time-consuming methods, such as gel electrophoresis and thin-layer chromatography, usually combined with radioisotope labeling (36,37). In this work, we sought to develop a high-throughput and easily accessible method for assessing the decapping activity and substrate profiling of various enzymes. To this end, we took advantage of fluorescent RNA aptamers, which are RNA constructs capable of binding strongly and specifically to small fluorogenic molecules (38,39). We synthesized a series of such RNAs capped with different structures using a standard *in vitro* transcription (IVT) reaction primed by appropriate di-/trinucleotide cap analogs. Then, we used these capped aptamers to design a fluorescence intensity (FLINT)-based assay and comprehensively studied the activity of multiple decapping enzymes against RNAs capped with various canonical and non-canonical structures (Figure 3). We also performed kinetic studies of the decapping process and demonstrated the potential of our assay to evaluate inhibitors of a viral decapping enzyme.

## Materials and methods

### General information

The starting materials necessary for chemical syntheses were obtained from commercial sources. All organic solvents that were used in the chemical syntheses under anhydrous conditions: dimethyl sulfoxide (DMSO, Honeywell, HPLC grade), N, N-dimethylformamide (DMF, anhydrous, Sigma-Aldrich), and acetonitrile (ACN, HPLC grade, J.T.Baker), were additionally dried over 4A molecular sieves. Other solvents: diethyl ether (CHEMPUR, p.a.), acetone (CHEMPUR, p.a.), methanol (MeOH, J.T. Baker, HPLC grade) and acetic anhydride (CHEMPUR, p.a.) were used as received.

At each stage, the progress of the syntheses was monitored by reversed phase high pressure (or high performance) liquid chromatography (RP-HPLC) and low resolution MS analyses. The final compounds were purified on a Sephadex A-25 ion exchange column using a linear gradient of triethylammonium bicarbonate (TEAB) buffer. When the compound did not show satisfactory purity, it was subjected to purification by semi-preparative HPLC.

RP-HPLC was carried out on a Agilent system with UV-detection at 254 nm for the analysis and final purification of the compounds. A Gemini column (NX-C18, 150 mm x 4.6 mm, 3 μm) was used to analyze the reactions progress with a flow rate of 1 mL/min. The solution of 50 mM ammonium acetate (CH_3_COONH_4_) pH 5.9, and the mixture of 50 mM CH_3_COONH_4_, pH 5.9 and ACN (1/1, V/V) were used as buffers. HiCHROM C18, 150 mm × 10 mm, 5 μm, with a flow rate of 4.7 mL/min was used as semi-preparative column. Buffers for RP-HPLC were as follows: A: 50 mM CH_3_COONH_4_, pH 5.9, B: 50 mM CH_3_COONH_4_, pH 5.9 / MeOH, 1/1, V/V.

Solid-phase syntheses of dinucleotides were performed using ÄKTA Oligopilot plus 10 synthesizer (GE Healthcare).

DEAE Sephadex A-25 (HCO_3_^−^ form) was used for purification by ion exchange column chromatography. TEAB buffer in deionized water was used as the mobile phase in a linear gradient: 0–0.7 M TEAB for nucleoside monophosphate, 0–0.9 M TEAB for nucleoside diphosphate and 0–1.2 M TEAB for dinucleoside triphosphate. Collected fractions containing expected product were combined, and the purity was confirmed using spectrophotometry analyze at 260 nm and RP-HPLC. Each time, the products were isolated as triethylammonium salts after concentration on rotary evaporator, followed by several evaporations with ethanol (96% and 99%), ACN and finally lyophilization.

High resolution mass spectra (HRMS) were obtained by the use of a micromass LCT electrospray time of flight. All nuclear magnetic resonance spectra were recorded using Bruker Avance III HD 500 MHz spectrometer.

### Chemical synthesis of *in vitro* transcription primers

The following syntheses were carried out according to previously described procedures: GpppApG (2) (40), m^7^GpppApG (3) (41), m^7^GpppA_m_pG (4) (41), NADpG (6) (42) and FADpG (7) (43). Automatic solid-phase syntheses of dinucleotide *pApG* (9) and it's activation (synthesis of Im-pApG (7)) were carried out using the procedure applied in (42).

#### Synthesis of m_3_^2,2,7^GpppApG (5)

Compound (5) (m_3_^2,2,7^GpppApG) was synthesized by a convergent approach to minimize the loss of yields in each step. The starting material was commercially available guanosine.

*Synthesis of 2-N,2-N,7-N-trimethylguanosine 5′-diphosphate* (10). The synthesis of compound (10) was carried out according to previously described procedure (44).

*Synthesis of m_3_^2,2,7^GpppApG* (5). Compound (7) as a sodium salt (141.03 mg, 0.20 mmol, 1 equiv.) was dissolved in DMSO (11 mL) and compound (10) as triethyl ammonium salt (148.62 mg, 0.31 mmol, 1.5 equiv.) was added. Dry MgCl_2_ (222.22 mg, 1.63 mmol, 8 equiv.) was then added, and the mixture was vigorously stirred at room temperature. The reaction was controlled by performing analyses on RP-HPLC every half hour. After 5 h, the reaction was terminated by adding an aqueous solution (110 mL) of disodium EDTA (606.51 mg, 1.63 mmol, 8 equiv.) and NaHCO_3_ (1/2 m EDTA) to adjust to pH 7. The compound (5) was purified using DEAE Sephadex A-25 ion-exchange column using a linear gradient of TEAB (0 – 1.2 M) followed by RP HPLC. The final synthesis product was lyophilized three times, and its structure was confirmed by high resolution MS and NMR. The product was obtained as a white powder in 43% yield.

**HRMS ESI (-**) calcd. m/z [M-H]^−^ C_33_H_44_N_15_O_24_P_4_^−^: 1158.16396, found: 1158.16535. **^1^H NMR (500 MHz, D_2_O, 25°C):***δ* = 9.09 (s, 1H, H8_m7G_), 8.49 (s, 1H, H2_A_), 8.30 (s, 1H, H8_A_), 8.05 (s, 1H, H8_G_), 5.99 (d, 1H, H1'_A_), 5.97 (d, 1H, H1'_m7G_), 5.81 (d, 1H, H1'_G_), 4.79 (m, 2H, overlapped with HDO, H3'_A_, H2'_G_), 4.71 (m, 1H, H2'_A_), 4.66 (m, 1H, H2'_m7G_), 4.53 (m, 1H, H4'_A_), 4.49 (m, 2H, H3'_m7G_, H3'_G_), 4.41–4.15 (m, 8H, H4'_m7G_, H4'_G_, H5'_A_, H5''_A_, H5'_m7G_, H5''_m7G_ H5'_G_, H5''_G_) 4.06 (s, 3H, N^7^-CH_3_), 3.12 (s, 6H, 2x N^2^-CH_3_) ppm; **^31^P NMR (202.5 MHz, D_2_O, H_3_PO_4_, 25°C):***δ* = 0.25 (s, 1P, P_AG_), -10.54 (m, 2P, P_α_, P_γ_), -21.97 (m, 1P, P_β_) ppm;

### Synthesis of GlcppUpG (8)

Synthesis was prepared as previously described in (43) with minor modifications. The reaction was carried out in DMSO instead of DMF. After 5 h, the reaction was terminated. The compound was purified on ion exchange chromatography (DEAE Sephadex) using a linear gradient of TEAB followed by a semi-preparative RP HPLC. The final synthesis product was lyophilized three times, and its structure was confirmed by high resolution MS and NMR. The results of the analyses are consistent with the literature (43). The product was obtained as a white powder in 81% yield.


\begin{eqnarray*}&& {\mathrm{HRMS \ ESI}}\left( - \right){\mathrm{calcd}}.\;{\mathrm{m}}/{\mathrm{z}}{\left[ {{\mathrm{M - H}}} \right]^ - }{\mathrm{C}_25}{\mathrm{H}_35}{\mathrm{N}_7}{\mathrm{O}_24}{\mathrm{P}_{3^ - }} \nonumber\\ && :910.09518,{\mathrm{found}}:910.09692.\end{eqnarray*}

### Protein expression and purification

Decapping and exoribonuclease protein (Mus musculus) – mDXO (Gene ID: 112403) sequence was obtained in plasmid vector (pETLGH_mDXO) thanks to Megerditch Kiledjian. mDXO protein (∼45 kDa) with Histidine tag at 5′ end was overexpressed in BL21 (DE3) RIL *E. coli* (Invitrogene) Procaryotic expression system. 6xHis-mDXO protein was induced by 0.5 mM IPTG solution at optical density 0.75 (bacterial culture) and bacteria were further cultured for 16 h at 18°C. Harvested cells were lysed in buffer containing: 20 mM Tris pH 7.5, 300 mM NaCl, 20 mM Imidazole, 5 mM β-Mercaptoethanol, 0.1 mg/mL lysozyme and mixture of protease inhibitors (Aprotinin, Leupeptin, Pepstatin, PMSF). The lysate was sonicated (15 min, 50% power, 15s on/off) and centrifuged. The supernatant was loaded on 2 × 5 mL HisTrap FF^TM^ column (Cytiva) equilibrated with a buffer: 20 mM Tris pH 7.5, 250 mM NaCl, 20 mM Imidazole, 5 mM β-Mercaptoethanol. 6xHis-mDXO protein was washed with buffer containing 1 M NaCl and eluted with buffer: 20 mM Tris pH 7.5, 250 mM NaCl, 300 mM Imidazole and 5 mM β-mercaptoethanol. Protein fractions were further purified on a Superdex 75 pg HiLoad 26/600 gel filtration column (Cytiva). Samples with mDXO protein concentrated to ∼15 μM, flash frozen and stored at -80°C in a buffer containing 20 mM Tris pH 7.5, 200 mM NaCl, 2 mM DTT, 10% glycerol.

Decapping complex of Dcp2-Dcp1 proteins (Gene ID: 167227) sequences were obtained in single plasmid vector (pET_PNRC_Dcp2/Trx_Dcp1) thanks to John Gross. Dcp2 protein with His-tag at 5′ end (∼48 kDa) and Dcp1 (∼21 kDa) were overexpressed simultaneously from a single vector in BL21 (DE3) RIL *E. coli* (Invitrogene) Procaryotic system. 6xHis-Dcp2/Dcp1 complex was induced with 0.4 IPTG at optical density 0.7 (bacterial culture), and the cells were further cultured for 16 h at 18°C. The culture was harvested and lysed in buffer: 50 mM NaH_2_PO_4_ pH 7.5, 500 mM NaCl, 20 mM imidazole, 1 mM DTT, 5% glycerol, 0.1 mg/ml lysozyme and mixture of protease inhibitors (Aprotinin, Leupeptin, Pepstatin, PMSF). The lysate was sonicated (15 min, 50% power, 15s on/off) and centrifuged. The supernatant with soluble fraction was loaded on 2 × 5 mL HisTrap FF^TM^ column (Cytiva) equilibrated with a buffer containing: 50 mM NaH_2_PO_4_ pH 7.5, 500 mM NaCl, 20 mM imidazole and 1 mM DTT, 5% glycerol. 6xHis-Dcp2/Dcp1 complex was washed with high salt buffer (1 M NaCl) and eluted with buffer: 50 mM NaH_2_PO_4_ pH 7.5, 500 mM NaCl, 500 mM imidazole and 1 mM DTT, 5% glycerol. Protein complex fractions were further diluted with buffer: 50 mM NaH_2_PO_4_ pH 7.5 and 1 mM DTT to achieve a salt concentration of 100 mM in the protein sample and then loaded on 5 ml HiTrap Heparin^TM^ column (Cytiva) equilibrated with buffer: 50 mM NaH_2_PO_4_ pH 7.5, 100 mM NaCl and 1 mM DTT, 5% glycerol. Dcp2/Dcp1 complex was eluted with high salt concentration buffer containing: 50 mM NaH_2_PO_4_ pH 7.5, 1 M NaCl, 1 mM DTT and 5% glycerol. The final purification step was gel filtration on a Superdex 75 pg HiLoad 26/600 gel filtration column (Cytiva). Samples with Dcp2/Dcp1 complex were collected and concentrated to ∼12 μM, flash frozen and stored at −80°C in a buffer: 50 mM HEPES, 150 mM NaCl and 1 mM DTT.

The mutant complex of Dcp2(E148Q)-Dcp1 proteins was expressed and purified as previously described (45).

Recombinant vaccinia virus mRNA decapping enzyme D9 (VACV D9) with additional C-terminal His-tag sequence was expressed and purified, as previously described (46).

Human scavenger decapping enzyme (hDcpS) was expressed and purified, as previously described (47).

Human decapping enzyme (hNudt16) was expressed and purified as previously described (48).

*Ec*RppH and 5′-Polyphosphatase were purchased from New England Biolabs (catalog number M0356S) and Lucigen (catalog number RP8092H), respectively.

### Design of aptamer probes

DNA oligonucleotide templates for RNA synthesis were designed with the goal of preventing any secondary structure formation at the 5′ end of the RNA probe (Supplementary Data, Supplementary Table S1). The 5′ cap structure was separated from the Broccoli aptamer by a 17-mer oligo(A) fragment, to ensure unperturbed cap recognition by the decapping enzymes. To minimize unwanted base pairing, we used the RNAfold WebServer as an *in silico* method of secondary structure prediction (Supplementary Data, Supplementary Figure S1) (49). Subsequently, the RNAComposer online tool was used for tertiary structure prediction (Supplementary Data, Supplementary Figure S4A) (50,51). Templates were then ordered from Genomed S.A., and the synthesis itself was carried out by Metabion International AG. Modifications at the 5′ end were introduced into the RNA sequence co-transcriptionally. RNA probe sequence (aptamer sequence in bold, underlined):

5′ GGGAAAAAAAAAAAAAAAAAGAGACGGUCGGGUCCA**GAUAUUCGUAUCUGUCGAGUAGAGUGUGGGCUCC** 3′.

### *In vitro* transcription of aptamer probes

RNA oligonucleotide (70 nt) aptamer probes were obtained by IVT using T7 RNA Polymerase from dsDNA templates. Each transcription reaction consisted of 1 μM oligonucleotide template DNA, 40 μl 5x Transcription Buffer (ThermoFisher Scientific), 5 mM NTPs (except for guanosine 5′-triphosphate (GTP), of which the concentration was 2 mM), 200 U RiboLock RNase inhibitor (ThermoFisher Scientific), 6 mM of respective cap analog, 20 mM MgCl_2_ and 20 μl of T7 RNA polymerase (1 mg/ml) to a total volume of 200 μl. The reaction was incubated for 4 h at 37°C, quenched and subsequently extracted by phenol-chloroform 1:1 mixture. RNAs were then purified using HPLC on Phenomenex Clarity Oligo-RP column in reverse-phase triethylamine acetate and acetonitrile system (Supplementary Data, Supplementary Figure S2A, B). RNA quality was verified using boronate affinity gel electrophoresis (APB-PAGE) (Supplementary Data, Supplementary Figure S3). Fractions thus obtained were then precipitated with ice-cold absolute ethanol and used in further experiments.

### RNA degradation by decapping enzymes and Xrn1

IVT products were separated into aliquots, each containing 2 μg of RNA dissolved in 20 μl of MQ water. In order to ensure complete unfolding of secondary structures, the samples were heated to 90°C for 2 min and then cooled down on ice for 2 min, as described in (52). Afterwards, the reaction buffer (NEBuffer 3: 100 mM NaCl, 50 mM Tris-HCl, 10 mM MgCl_2_, 1 mM DTT, pH 7.9; New England Biolabs) was added, alongside decapping enzyme solution to a final concentration of 50 nM. Samples were then incubated for 30 min in 37°C. Subsequently, 2 μl of Xrn1 (1 U per 1 μg of RNA, New England Biolabs) were added, and samples were further incubated for another 30 min.

### Aptamer-ligand complex formation

After stopping decapping reaction and Xrn1 enzymatic activity by heating the samples up to 90°C for 2 min and ice cooling (2 min), the folding buffer was added (40 mM Tris-HCl pH 7.5, 125 mM KCl, 5 mM MgCl_2_ and 10 μM DFHBI-1T). The final volume of each sample was 150 μl. The RNA refolding protocol was based on previous work with the Broccoli aptamer (52,53). Full procedure is described in the Supplementary Data.

### Fluorescence intensity measurements

Following the refolding procedure, samples were transferred onto a 96-well black microplate (Greiner Bio-One). FLINT readouts were performed using Synergy H1 Microplate Reader (BioTek) at room temperature (RT), at excitation wavelength of 472 nm and emission wavelength of 507 nm (Supplementary information, Supplementary Figure S4B).

### Validation by boronate affinity electrophoresis

After enzymatic degradation and fluorescence emission measurements, the samples were further examined by use of boronate affinity electrophoresis (44). Each experiment was analyzed and validated by assessment of band intensity and migration distance from a corresponding RNA sample (Supplementary Data, Supplementary Figure S4E). Around 1% v/v of APB (3-acrylamidophenylboronic acid) was added to a 20% solution of acrylamide/bisacrylamide (19:1) and incubated in 65°C until fully solubilized. Afterward, the solution was cooled to RT and diluted to 15% of acrylamide and 1×Tris-Borate-EDTA buffer (TBE). Ammonium persulfate and TEMED (N,N,N’,N’-tetramethylethane-1,2-diamine) were added, and the solution was allowed to polymerize. After electrophoresis, gels were stained with SYBR Gold (Invitrogen) and visualized using Typhoon FLA 9500 biomolecular imager (GE Healthcare).

### Kinetic decapping assay

For assessment of the Michaelis–Mnten kinetics, 50 nM of hDcp2/Dcp1 was incubated with the reaction substrate, 5′ cap-1 RNA-probe (4) at four time points: 0, 15, 30 and 60 min and subsequently treated for 30 min with 1 U Xrn1 per μg RNA in the reaction buffer (NEBuffer 3). The following substrate concentrations (μM) were used: 0, 0.6, 0.8, 1, 1.5, 2, 3 and 5. After each time point, the reaction was immediately terminated by heating at 90 °C for 5 min. In the next step, the formation of the aptamer–ligand complex and the measurement of the FLINT were carried out, the procedures for which have been described above (Materials and methods, sections ‘Aptamer-ligand complex formation’ and ‘Fluorescence intensity measurements’). The initial reaction rates were derived from the relative decrease in FLINT by fitting a linear regression equation to each substrate concentration, compared to the control sample treated with Xrn1 alone (without Dcp2) and plotted against the initial RNA concentration (Figure 7C). The reaction rates were determined at four timepoints: 0, 15, 30 and 60 min. (GraphPad Prism). Fitting of the Michaelis–Menten equation (55,56) to this data set provided kinetic parameters of the studied decapping reaction:


\begin{eqnarray*}v = \;\frac{{{V_{max}}\left[ S \right]}}{{{K_M} + \left[ S \right]}}\end{eqnarray*}

where V_max_ – maximum rate of reaction (horizontal asymptote), K_M_ – substrate concentration at 50% of V_max_, [S] – substrate concentration. At maximum rate, V_max_ = k_cat_[E]_0_, where k_cat_ is the turnover number, i.e. maximum number of molecules per enzyme molecule per unit time and [E]_0_ is the initial enzyme concentration.

## Results and discussion

### Probe design and synthesis

**Figure 4.**
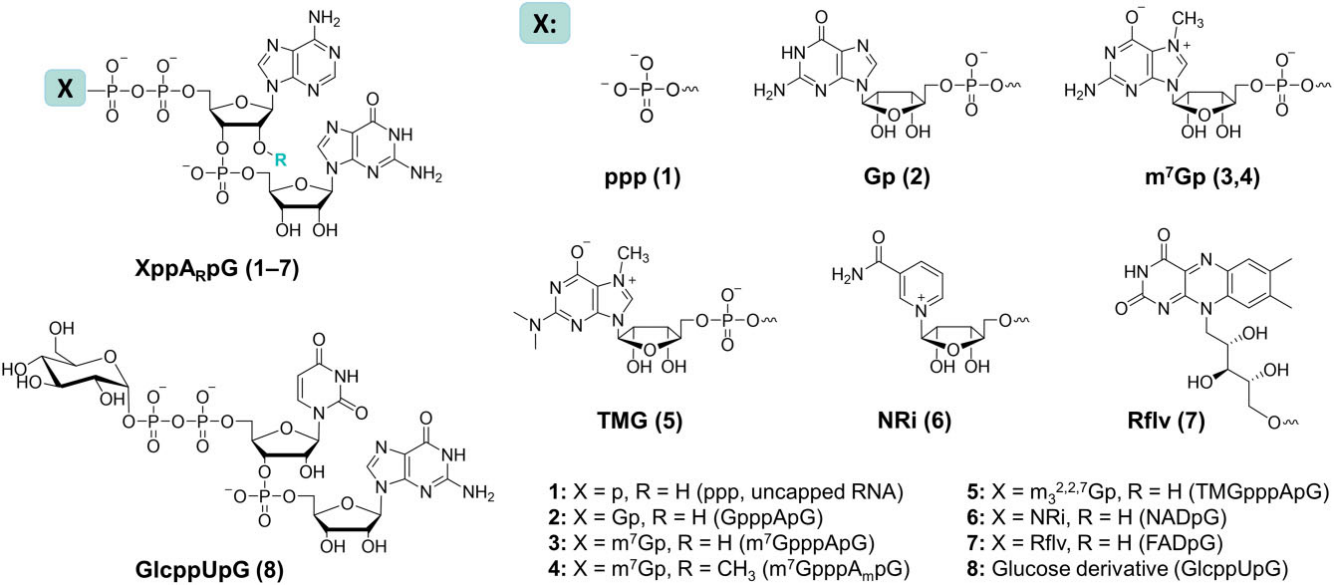
Chemical structures of di/tri-nucleotide cap analogs used in this work as IVT primers.

Our goal was to design universal molecular probes for simple and quantitative assessment of RNA decapping activity of various enzymes. It is critical that the 5′ ends of these RNA probes resemble natural RNAs as closely as possible and do not bias their affinity for decapping enzymes. Therefore, we focused on fluorogenic RNA aptamers that can be selectively degraded after decapping and enable tracking of substrate concentration changes. All RNAs were synthesized by IVT reaction using T7 RNA Polymerase and a DNA template encoding the Broccoli aptamer (53). To minimize the secondary structure formation near the 5′ end of RNA, which might have interfered with cap hydrolysis, we introduced a 20-nt spacer between the Ф6.5 promoter and the aptamer coding sequence. Secondary structure prediction of the resulting 70-nt RNA (RNAfold WebServer (49) and RNAComposer (50,51)) revealed a single-stranded fragment at the 5′ end that was unlikely to fold into a higher order structure (Supplementary Figure S4A). To prepare the RNA probes with different cap variants, we designed and synthesized a series of IVT primers based on di- or tri-nucleotide XppA_R_pG structures (Figure 4), which ensure high capping yields and correct cap orientation (43,57,58). The synthetic pathways for most of the cap analogs involved solid-phase synthesis of pA_(m)_pG dinucleotides by the phosphoramidite method, their subsequent activation into *P*-imidazolides, (Supplementary information, Supplementary Scheme S1) followed by a Mg(II)- or Zn(II)-mediated coupling reaction with an appropriately modified guanosine diphosphate derivative (GDP, m^7^GDP, TMGDP, or Rflv-p) (41,43). In the case of the NAD-cap primer, a fragment derived from nicotinamide monophosphate was activated instead of the dinucleotide (42). The IVT primer for UDP-glucose-capped RNA was designed as a GlcppUpG (8) structure (Figure 4) and synthesized analogously using glucose 1-phosphate and activated pUpG dinucleotide (43). Each cap analog was added to the IVT reaction mixture in 3-fold excess over GTP to achieve high capping efficiencies (>90%) and satisfactory RNA yields (1.4–1.8 μg/μL of reaction mixture). The uncapped (i.e. 5′-triphosphorylated) RNAs were separated from the capped RNAs by RP-HPLC (Supplementary information, Supplementary Figure S2A, B) and the quality of each transcript was verified by TBE PAGE (Supplementary information, Supplementary Figure S3). The observed heterogeneity of the RNAs (the bands above and below the main band) results from insertions of untemplated nucleotides by polymerase and abortive termination of the transcription reaction (59), but we expected that it should not significantly affect the decapping rate.

### Validation of RNA aptamer probes

**Figure 5.**
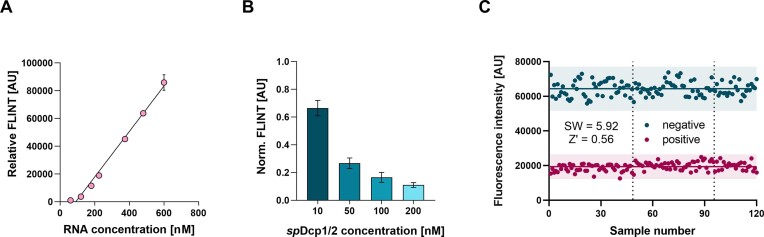
(**A**) The FLINT of the refolded aptamer in the presence of a fluorogenic ligand DFHBI-1T is proportional to the RNA concentration. (**B**) FLINT correlates negatively with increasing decapping enzyme concentration. (**C**) The determination of signal window and Z’ factor.

First, we verified that the FLINT (exc. 472 nm, em. 507 nm) of the refolded aptamer in the presence of a fluorogenic molecular rotor DFHBI-1T is proportional to the RNA concentration (Figure 5A). We also found that the concentration of DFHBI-1T does not strongly affect the FLINT as long as it is present in excess relative to RNA (Supplementary information, Supplementary Figure S4D). To evaluate the designed RNA sequence as a decapping probe, we incubated the cap-0-RNA probe (3) with *Sp*Dcp2 at different enzyme concentrations for 30 min and then with the Xrn1 5′-exonuclease for another 30 min to completely degrade the decapped transcripts (Figure 5B). A variety of incubation times for the decapping enzyme and the RNA were tested and the results are shown in Supplementary Figure S4C (Supplementary information). Next, the sample was slowly annealed in the presence of DFHBI-1T to refold the remaining intact (i.e. not decapped and thus not degraded by Xrn1) RNAs into a fluorescent complex (53,60). Since the decapped RNAs are degraded by Xrn1 and thus cannot form aptamers, the observed fluorescence signal is negatively proportional to the decapping activity of the tested enzyme. As expected, we observed an increase in *Sp*Dcp2 activity with increasing enzyme concentration (Figure 5B), although the correlation was not linear because the substrate/enzyme ratio changed significantly during the reaction. Further tests using RNA probes with other caps showed that in order to differentiate the enzyme activity toward specific cap structures, the reaction should be carried out with an excess of RNA over the enzyme (Figure 5A,B). We found that the optimal substrate and enzyme concentrations, which provide the maximum signal-to-noise ratio at the lowest possible synthetic cost, are 600 nM RNA and 50 nM enzyme (12-fold excess) (53,60). The quality of the optimized decapping assay was assessed to verify its suitability for screening studies. The cap-0-RNA probe (3) was incubated with either the hDcp2/Dcp1 complex (positive samples) or its catalytically inactive mutant E148Q (negative samples) in multiple replicates (120 each), followed by Xrn1 treatment and RNA refolding in the presence of DFHBI-1T. The FLINT was quantified using a microplate reader, and the statistical analysis was performed (Figure 5C) (61). The dispersion of the data points was relatively low for both positive and negative samples (coefficient of variation CV for each plate in the range of 11–12% for positive samples and 6–7% for negative samples), and no signs of systematic sources of variability were observed, both within and between the plates. The signal window (SW = 5.92) and Z’ factor (0.56) indicate that our assay is a reliable method for screening the activity of decapping enzymes.

### Method applications

#### Substrate profiling of decapping enzymes

**Figure 6.**
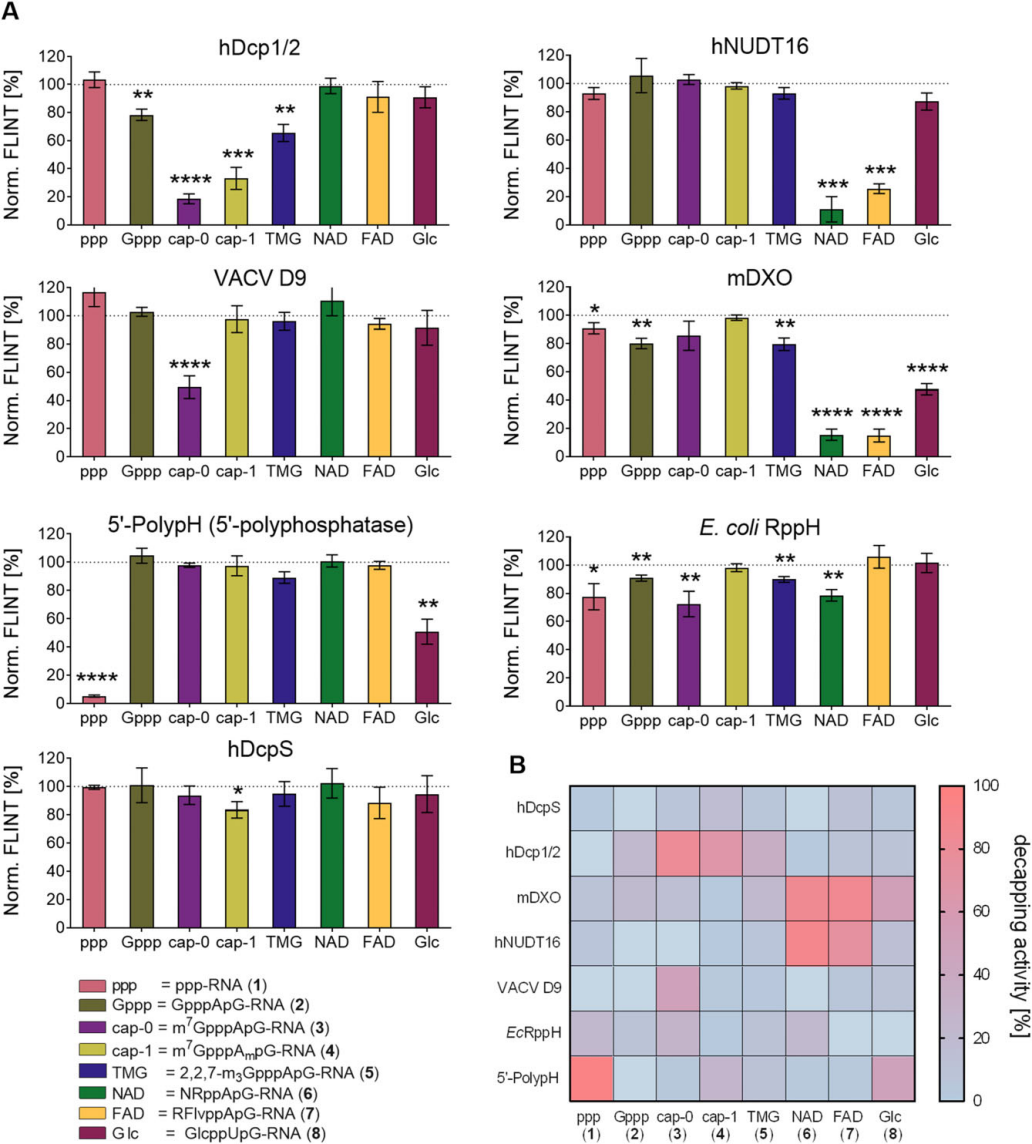
(**A**) Normalized FLINT profiles of each examined cap degradation enzyme. Bars are percentage ratio of fluorescence at 507 nm of 10 μM DFHBI-1T, 0.6 μM RNA, 2U Xrn1, 50 nM decapping enzyme to negative control: 10 μM DFHBI-1T, 0.6 μM RNA and 2U Xrn1 (no decapping enzyme). Every bar represents the mean ± SD of at least three independent experiments. Statistical significance: no symbol – not significant, * – *P* < 0.05, ** – *P* < 0.01, *** – *P* < 0.001, **** – *P* < 0.0001 (t-test). (**B**) Heat map of seven decapping enzymes versus eight RNA probes capped with 5′ cap analogs. Values are corresponding capped RNA degradation normalized to negative control (Xrn1 only). 100% represents full RNA degradation (i.e. no fluorescence); 0% represents results comparable to the negative control.

The optimized assay was then used to examine phosphohydrolytic activity of seven different proteins (human Dcp2 in complex with Dcp1, human DcpS, human NUDT16, mouse DXO, *E. coli* RppH, Vaccinia virus D9, and *E. coli* 5′-polyphosphatase) against seven different cap structures and the 5′-triphosphate (Figure 6). Consistent with previous reports (53–55), hDcp2-Dcp1 complex exhibited robust activity predominantly towards canonical cap-0 and cap-1 structures and a moderate activity towards unmethylated GpppA structure and the TMG cap (Figure 6A) (62–64), while the uncapped RNA (pppG-RNA) and RNAs with non-canonical caps (NAD, FAD, UDP-Glc) remained intact (18,33,62–66). Another member of the Nudix family, hNUDT16, showed substantial degradation of both NAD and FAD caps without significant activity on other capped RNAs. This is in contrast to previous reports, as hNUDT16 has been shown to be able to decap both U8 snoRNA (with TMG cap) and mRNA (with m^7^G cap) *in vitro* (66,67). This difference may be due to the fact that hNUDT16 requires divalent Mn^2+^ or Co^2+^ ions to cleave m^7^G structures (48), which were not present in our assay. Our conditions (with Mg^2+^), which seem to be more biologically relevant, suggest a preference for metabolite-like structures, which is consistent with recent studies on this enzyme (68). Interestingly, vaccinia virus protein D9 showed moderate activity only on cap-0 substrates, with very little or no activity on the other cap structures tested. To our knowledge, this enzyme has not been previously studied on RNAs with cap structures other than m^7^G (69–71). The bacterial Nudix phosphohydrolase, RppH, showed multiple activities, none of which exceeded 30% decapping under the conditions used in the assay. mDXO, considered to be part of the pre-mRNA quality control (72), degraded predominantly non-canonical 5′ RNA ends (NAD, FAD and UDP-Glc), but surprisingly, showed only a very weak activity towards aberrant GpppA cap, comparable to that observed for TMG cap (22,73). As expected for the scavenger decapping enzyme hDcpS, which acts only on very short RNA substrates, we observed little to no degradation of all transcripts tested (24). Finally, we tested a commercially available recombinant 5′-polyphosphatase, which is commonly used to hydrolyze 5′-triphosphates to 5′-monophosphates. It was indeed robust on 5′-triphosphate RNA and showed moderate activity on 5′-glucose-1-phosphate-capped RNA. To directly compare the results obtained for different enzymes, we visualized the data as a heat map (Figure 6B).

All decapping experiments were independently validated using boronate affinity electrophoresis (49), in which standard 15% polyacrylamide gel was copolymerized with 1%_v/v_ acryloylaminophenyl boronic acid, providing better separation of capped vs uncapped RNA bands (Supplementary information, Supplementary Figures S5–S12) (54). The results from PAGE analyses were in good agreement with the data obtained from our aptamer-based FLINT decapping assay, confirming its reliability.

To test our assay in a more challenging biological system, we monitored the degradation of cap-0-RNA (3) and FADpG-RNA (7) in HEK293F cell extracts (Supplementary Figure S13A). We found that the RNA probes undergo fast degradation in the extracts (samples E) to a degree comparable to that of recombinant enzymes (samples C), even if no Xrn1 was added (samples F). At this point, it remains unclear whether the RNA probes are degraded via a process involving decapping and 5′ exonucleolytic cleavage, or whether an alternative mechanism is responsible. However, PAGE analysis confirmed that the RNA probes incubated with cell extracts were indeed hydrolyzed (Supplementary Figure S13B).

#### Characterization of decapping inhibitors and competitors

**Figure 7.**
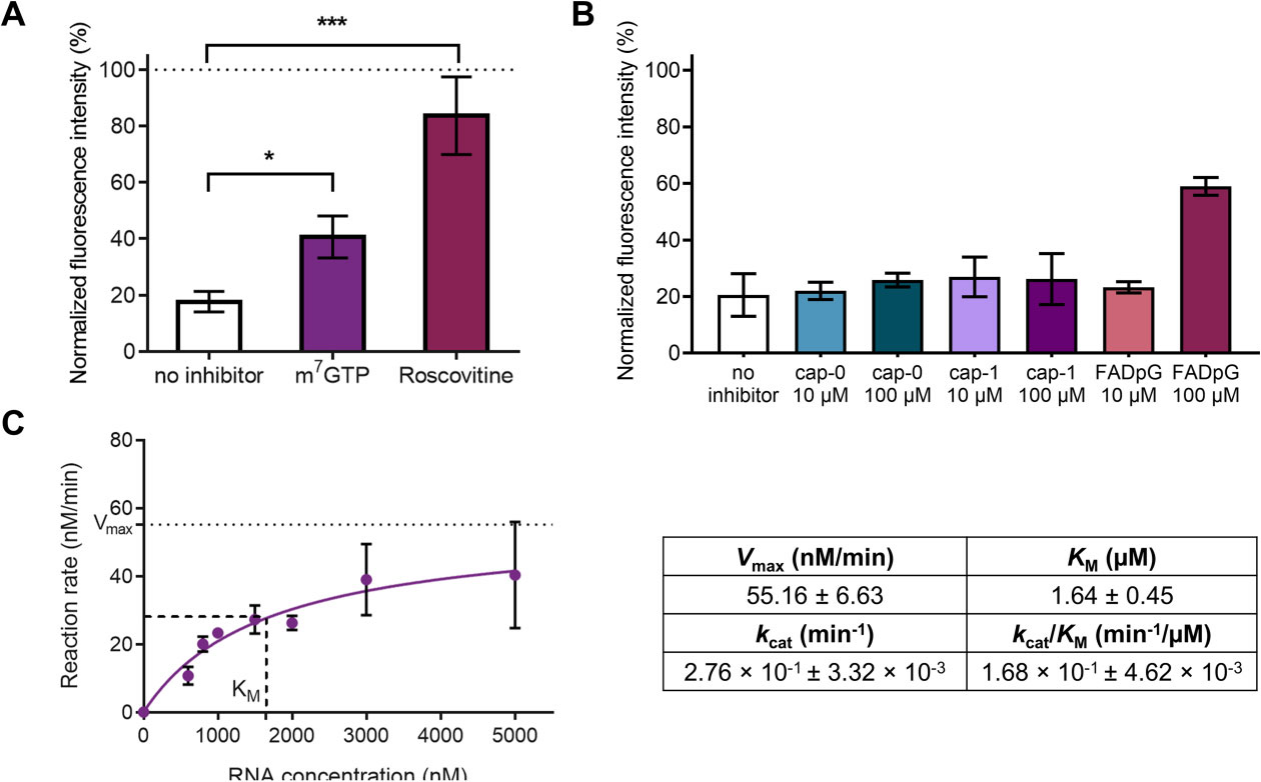
(**A**) Normalized FLINT of 200 nM VACV D9 incubated with Xrn1 and 1 mM of respective inhibitor. Fluorescence level of cap-0 (3) RNA (600 nM) incubated with 200 nM D9 and Xrn1 without any inhibition is shown in white. Every bar represents the mean ± SD of at least three independent experiments. Statistical significance: no symbol – not significant, * – *P* < 0.05, ** – *P* < 0.01, *** – *P* < 0.001, **** – *P* < 0.0001 (t-test). (**B**) Normalized FLINT of 50 nM hDcp2/Dcp1 enzyme incubated with 2U Xrn1, 600 nM cap-0-RNA probe (3) and in the presence of cap-0, cap-1, or FAD-cap at concentrations of 10 or 100 μM. (**C**) hDcp2-RNA reaction rate as a function of cap-1 RNA (4) concentration, fitted with Michaelis–Menten model equation (GraphPad Prism). The obtained kinetic parameters are shown.

After profiling the activity of various enzymes, we decided to test whether our assay is suitable for characterizing the potency of decapping inhibitors. To this end, we chose the D9 decapping enzyme, which showed high specificity for the cap-0 structure and, as a viral protein, could be considered as a model therapeutic target. We tested two compounds that were identified as hits in a previously reported study (74), namely 7-methylguanosine 5′-triphosphate (m^7^GTP) and a cyclin-dependent kinase inhibitor roscovitine (Seliciclib). Consistent with previous results, significant levels of D9 inhibition were observed for both compounds (around 80% for roscovitine and 40% for m^7^GTP; Figure 7A), confirming the utility of our assay for screening inhibitors of decapping enzymes.

An analogous approach can be used to study the recognition of free cap structures (e.g. products of RNA 3′→5′ degradation) by decapping enzymes, which cannot be directly monitored by our assay. To demonstrate this, we assayed the hDcp2/Dcp1 enzyme with the cap-0-RNA probe (3) in the presence of cap-0, cap-1 or FAD-cap at concentrations of 10 or 100 μM (Figure 7B). Only FADpG at 100 μM (ca. 170-fold excess over RNA), decreased the decapping rate, while for the samples, no significant competition between free cap structures and the RNA probe was observed, which is consistent with previous reports on Dcp2 activity (75).

#### Decapping kinetics studies

Next, we attempted to adapt our assay to monitor the kinetics of *in vitro* decapping. To this end, we performed a time-resolved quantification of RNA cap-1 hydrolysis by the human Dcp1-Dcp2 complex. Cap-1 RNA probe (4) at concentrations ranging from 0.6 to 5 μM was subjected to decapping by Dcp2 for 60 min, followed by Xrn1 treatment, refolding in the presence of DFHBI-1T and fluorescence readout. Our results report lower *k*_cat_ values than most of the previous studies on the yeast *Sp*Dcp1-Dcp2 decapping complex using a single-turnover kinetics analysis (75–77). This could be attributed to the differences in the enzyme origin (human vs. yeast), length and secondary structure of the RNA substrates, type of the 5′-cap (cap-0 versus cap-1), and different measurement buffers. Nevertheless, the obtained value is significantly closer to the *k*_cat_ reported for m^7^GpppG-RNA_29_ (*k*_cat_ = 2.2 min^−1^) than for the GpppG-RNA_29_ (*k*_cat_ = 0.0012 min^−1^) decapping by *Sp* GB1-Dcp1-Dcp2(1–245) (75).

## Conclusions

RNA decapping is one of the most important processes contributing to RNA stability and thus to the regulation of gene expression. A number of genetic diseases have been linked to disruptions in this process. Recently, a variety of non-canonical 5′ cap structures, including abundant cofactors and metabolites such as NAD, FAD and UDP-glucose, have been identified in different organisms. These findings call for a re-evaluation of the substrate specificity of numerous proteins that have been reported to exhibit decapping activity. Unfortunately, the methods currently used for this purpose are costly and time-consuming, making them particularly inefficient for comparative studies. To address this problem we developed a FLINT assay that allows high-throughput monitoring of RNA decapping reactions.

First, we synthesized a series of di- and tri-nucleotide cap analogs, from canonical m^7^Gppp- structures and aberrant Gppp- cap to modified biological cofactors and metabolites such as NAD, FAD or UDP-glucose. Next, we used them all as *IVT* primers to produce capped 70-nucleotide fluorescent probes, based on the Broccoli RNA aptamer sequence. We exposed these probes to various phosphorohydrolytic proteins, including several decapping enzymes, and assessed their activity by fluorescence measurements. This comprehensive approach allowed us to profile and directly compare the substrate specificity of these enzymes. Our results confirmed the previous literature reports on Dcp2, DcpS and 5′-polyphosphatase but also shed some new light on the activity of hNUDT16, mDXO and VACV D9 enzymes. We have also demonstrated the utility of RNA FLINT probes for evaluating and characterizing the kinetic properties of inhibitors of decapping enzymes. Our assay is fully compatible with a microplate format, making it a good candidate for high-throughput screening of decapping inhibitors. We hope that this multi-purpose tool will contribute to the flourishing field of RNA biology and help to better understand the intricate processes of RNA degradation mechanisms, which are critical for both the natural function of RNAs and their therapeutic applications.

## Supplementary Material

gkae919_Supplemental_File

## Data Availability

All raw data are available from the corresponding author upon reasonable request. RNAfold web server is a part of the Vienna RNA Websuite: a free, open access web interface for *in silico* folding, design and analysis of RNA sequences (49). RNAComposer is an online web-based, fully automated RNA structure modeling server, accessible through http://rnacomposer.ibch.poznan.pl and https://rnacomposer.cs.put.poznan.pl/.
