# Supplementary material for: Aptamer-based assay for high-throughput substrate profiling of RNA decapping enzymes
Source: Nucleic Acids Res. 2024 Oct 24;52(21):e100. doi: 10.1093/nar/gkae919 (PMC11602136; doi:10.1093/nar/gkae919)
Supplement: gkae919_Supplemental_File [file gkae919_supplemental_file.docx]

Supplementary Data

**Aptamer-based assay** **for high-throughput substrate profiling of RNA decapping enzymes**

Katarzyna Grab^1,2^†, Mateusz Fido^1^† ,Tomasz Spiewla^1,2^, Marcin Warminski^1^, Jacek Jemielity^3^, Joanna Kowalska^1,^*

^1^ Division of Biophysics, Institute of Experimental Physics, Faculty of Physics, University of Warsaw, Pasteura 5, 02-093 Warsaw

^2^ Doctoral School of Exact and Natural Sciences, University of Warsaw, Żwirki i Wigury 93, 02-089 Warsaw

^3^ Centre of New Technologies, University of Warsaw, Banacha 2c, 02-097 Warsaw

* To whom correspondence should be addressed. Email: [jkowalska@fuw.edu.pl](mailto:jkowalska@fuw.edu.pl)

† Joint First Authors

Contents

[I. DNA and RNA preparation 2](#_Toc173439518)

[II. RNA refolding protocol 4](#_Toc173439519)

[III. Optimisation of measurement conditions 4](#_Toc173439520)

[IV. Activity profiles and APB-PAGE for all assayed cap degradation proteins 5](#_Toc173439521)

[1. *Mus musculus* decapping exoribonuclease (mDXO) 6](#_Toc173439522)

[1. Human decapping protein complex (hDcp1/2) 6](#_Toc173439523)

[2. Human decapping scavenger protein (hDcpS) 7](#_Toc173439524)

[3. Human Nudix Hydrolase 16 (hNUDT16) 8](#_Toc173439525)

[4. Vaccinia virus decapping protein D9 (VACV D9) 9](#_Toc173439526)

[5. *Escherichia coli* pyrophosphohydrolase (*Ec*RppH) 11](#_Toc173439527)

[6. 5’-polyphosphatase (5’-PolyPH) 12](#_Toc173439528)

[7. Evaluation of the potential for use of the FLINT assay in cell extracts 14](#_Toc173439529)

[2. Chemical syntheses of cap structures 14](#_Toc173439530)

[3. Chemical structure, HRMS spectrum, NMR spectrum and HPLC chromatogram of synthesized new compound 15](#_Toc173439531)

[REFERENCES 19](#_Toc173439532)

# DNA and RNA preparation

**Table S1.** DNA template sequences used for aptamer probe synthesis:

DNA Starters (Φ6.5 T7 RNAP promoter):

| coding strand (90 nt) | CAGTAATACGACTCACTATAGGGAAAAAAAAAAAAAAAAAGAGACGGTCGGGTCCAGATATTCGTATCTGTCGAGTAGAGTGTGGGCTCC |
| --- | --- |
| template strand (90 nt) | GGAGCCCACACTCTACTCGACAGATACGAATATCTGGACCCGACCGTCTCTTTTTTTTTTTTTTTTTCCCTATAGTGAGTCGTATTACTG (90) |

The first transcribed nucleotide has been underlined.

RNA Nucleotide Sequence of the Φ6.5-poliA-Broccoli probe (Broccoli aptamer sequence highlighted in **green**):

GGGAAAAAAAAAAAAAAAAAGAGACGGUCGGGUCCA**GAUAUUCGUAUCUGUCGAGUAGAGUGUGGGCUCC** (70)


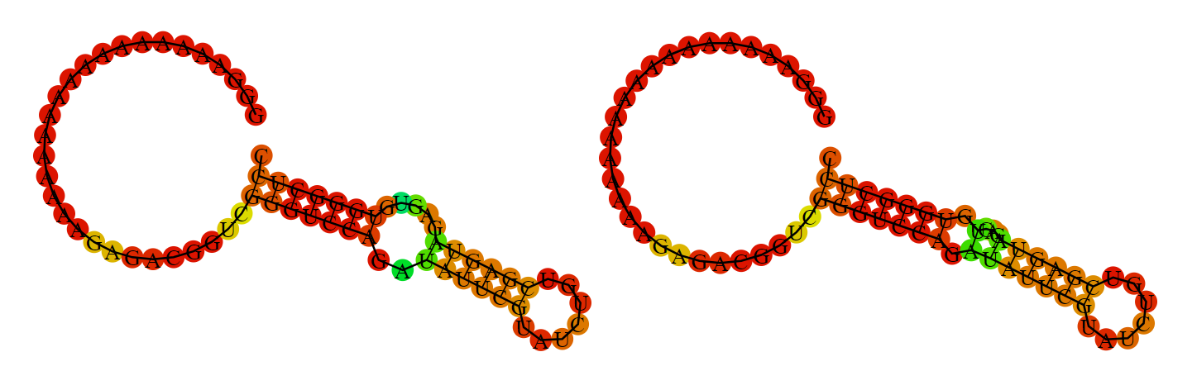


**Figure S1.** Predicted RNA probe secondary structure (RNAFold WebServer (1)).

**A**


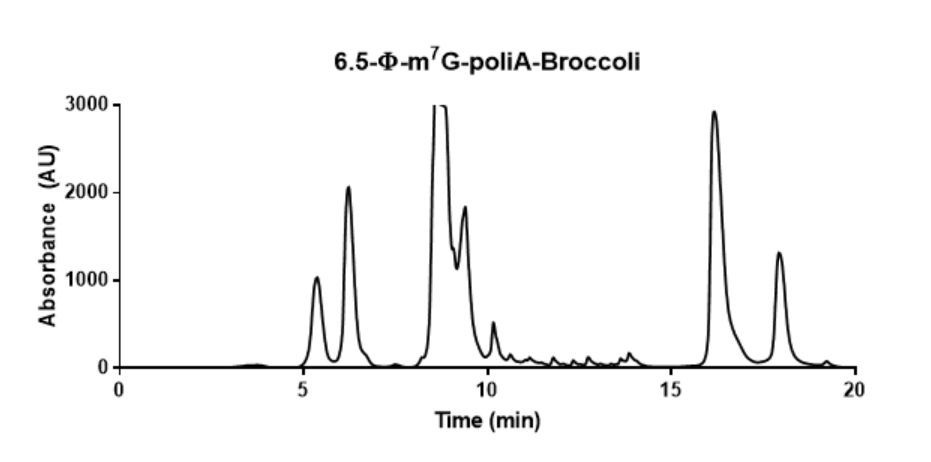


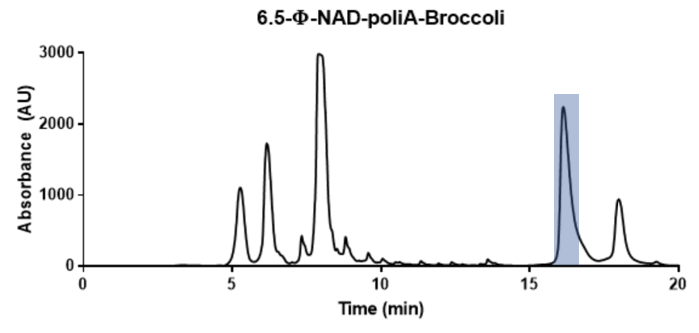


**B**

**Figure S2.** Example RP-HPLC profiles from the purification of aptamer probes capped with

m^7^GpppApG (**A**) and NADpG (**B**). The collected fractions are marked by the blue rectangle.


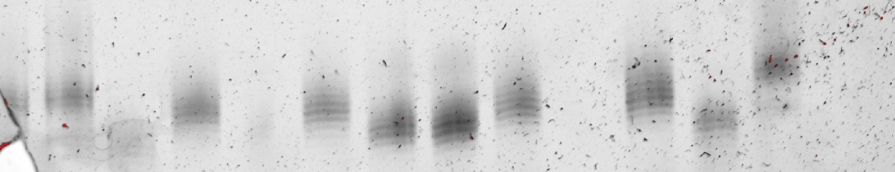


Capped RNA

Uncapped RNA


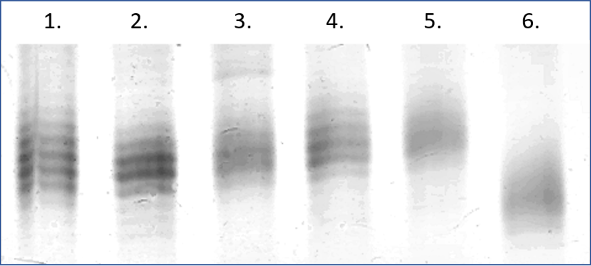


Uncapped RNA

Capped RNA

1. m^7^GpppApG-poliA-Broccoli
2. m^7^GpppA_m_pG-poliA-Broccoli
3. NRppApG-poliA-Broccoli
4. TMGpppApG-poliA-Broccoli
5. GpppApG-poliA-Broccoli
6. 5’ ppp-poliA-Broccoli

**Figure S3.** Boronate affinity gel electrophoresis (APB-PAGE) of capped RNAs obtained by IVT. Example of several aptamer probes with canonical and non-canonical caps at the 5'-end and a probe containing triphosphate at the 5'-end of the RNA are shown on the gel.

# RNA refolding protocol

RNA aptamer refolding protocol was based on “slow-cooling” approach outlined in (Okuda, et al. 2017) (2). Full procedure, adapted to our laboratory conditions:

- 1. Incubation of RNA samples for 2 minutes in 90°C
  2. Cooling down of the samples for 2 minutes on ice
  3. Addition of the refolding buffer (40 mM Tris-HCl pH 7.5, 125 mM KCl)
  4. Addition of MgCl_2_ (C_f_ MgCl_2_ = 5 mM)
  5. Addition of the DFHBI-1T ligand (C_f_ DFHBI-1T = 10 µM)
  6. Heating the samples up to 65°C
  7. Slowly cooling down from 65°C to 25°C at a rate of 1°C/min
  8. Transfering samples from sterile Eppendorf tubes onto a black 96-well Greiner polysterene microplate
  9. Fluorescence intensity measurements: λ_exc_ = 472 nm, λ_em_ = 507 nm @ RT

# Optimisation of measurement conditions


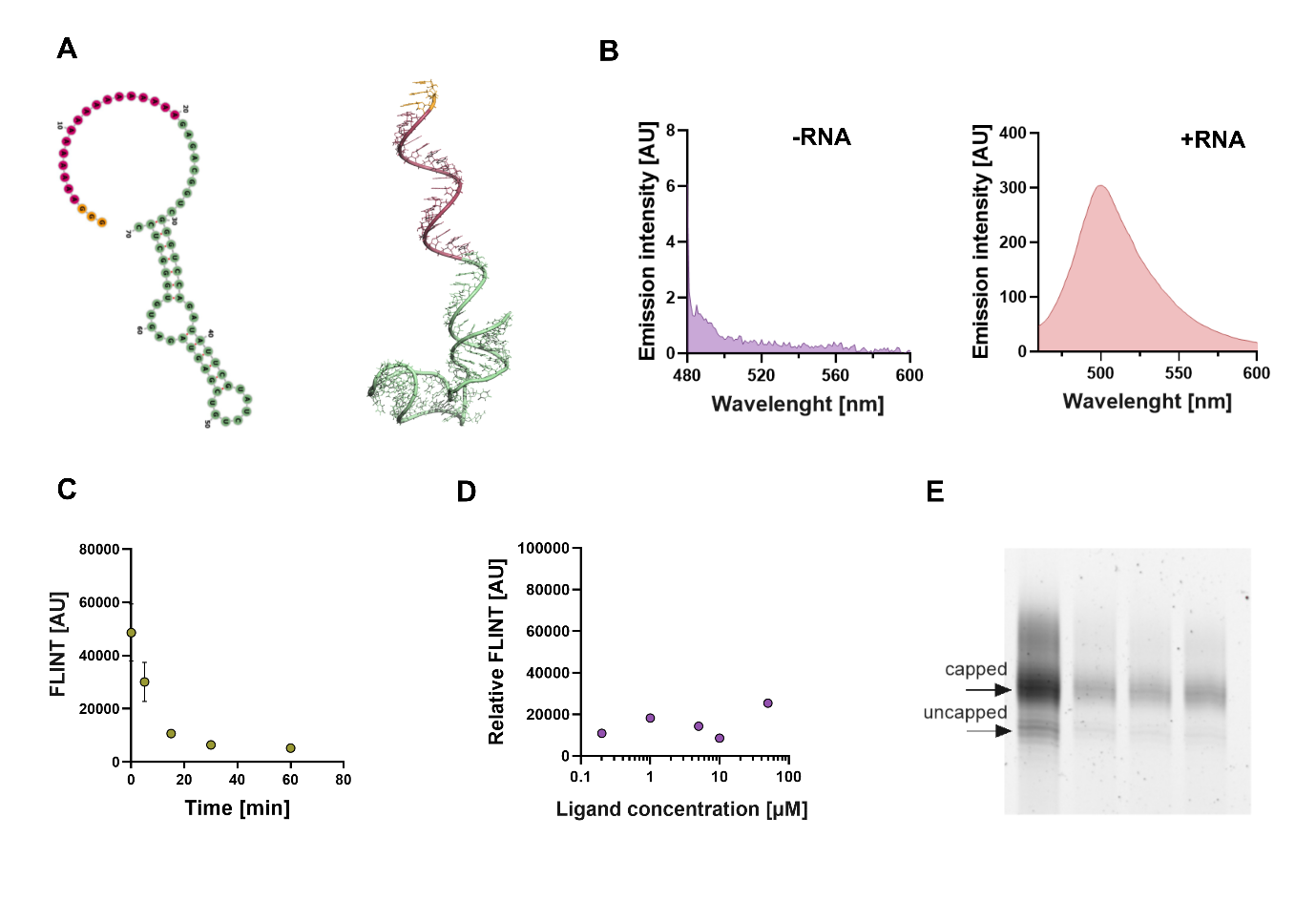


**Figure S4.** (**A**) Predicted minimum free energy (left) and centroid (right) RNA secondary structure. Broccoli aptamer sequence is highlighted in green. Graphical output taken from Vienna RNA Websuite forna web server. The spacer present at 5’ end of the probe (red) limits local base pairing. PyMOL visualization of predicted tertiary structure output from RNAComposer, colored correspondingly. (**B**) 10 µM buffer solution of the DFHBI-1T fluorophore does not exhibit any significant fluorescence emission. Upon adding 200 nM of uncapped RNA aptamer probe and refolding, a large increase in fluorescence intensity (peak 507 nm) is observed. (**C**) Changes in fluorescence intensity during incubation of 2 µg cap-0-polyA-Broccoli (**3**) probe with 50 nM hDcp2/Dcp1 enzyme. (**D**) Emission does not correlate with DFHBI-1T concentration. (**E**) Gel image of boronate affinity electrophoresis experiment containing four cap-0 RNA aptamer probe (**3**) replicates and 1% APB to polyacrylamide v/v. Visible separation between capped and uncapped RNA bands can be observed.

# Activity profiles and APB-PAGE for all assayed cap degradation proteins

## *Mus musculus* decapping exoribonuclease (mDXO)


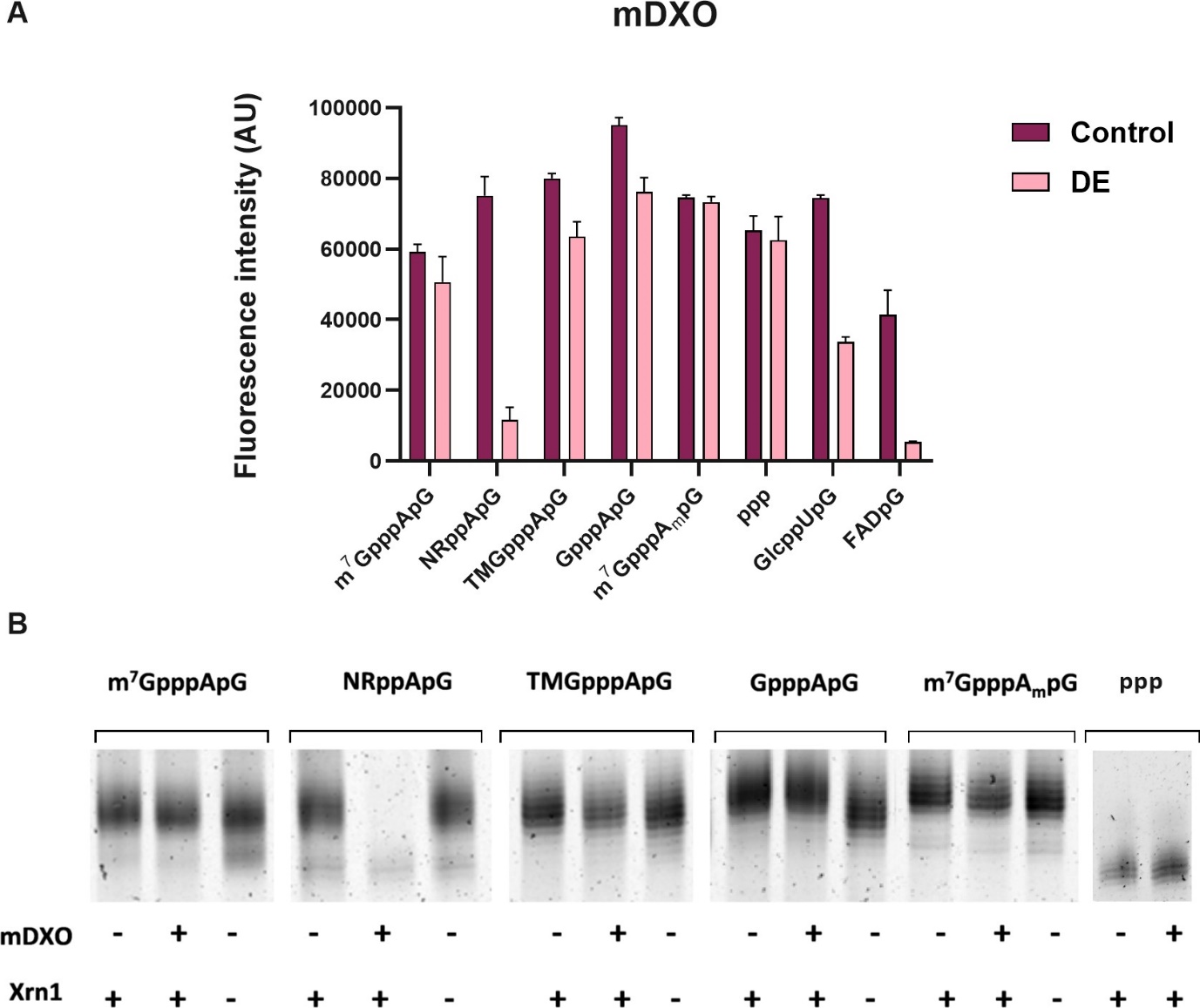


**Figure S5.** (**A**) Relative fluorescence intensity graphs for all aptamer probes used in this work. **Control**: 2 µg of capped RNA probe incubated with 2U Xrn1 for 30 minutes in 37°C. **DE** **(decapping experiment)**: 2 µg of capped RNA probe incubated with 50 nM mDXO for 30 minutes in 37°C and then with 2U Xrn1 for 30 minutes in 37°C. Every bar is SD of at least 3 independent experiments. Gel images for GlcppUpG- and FADpG- RNAs are shown in Figure S12. (**B**) Validation by boronate affinity electrophoresis (APB-PAGE) of the decapping FLINT assay with 50 nM mDXO and 2 µg of co-transcriptionally capped RNA.

## Human decapping protein complex (hDcp1/2)


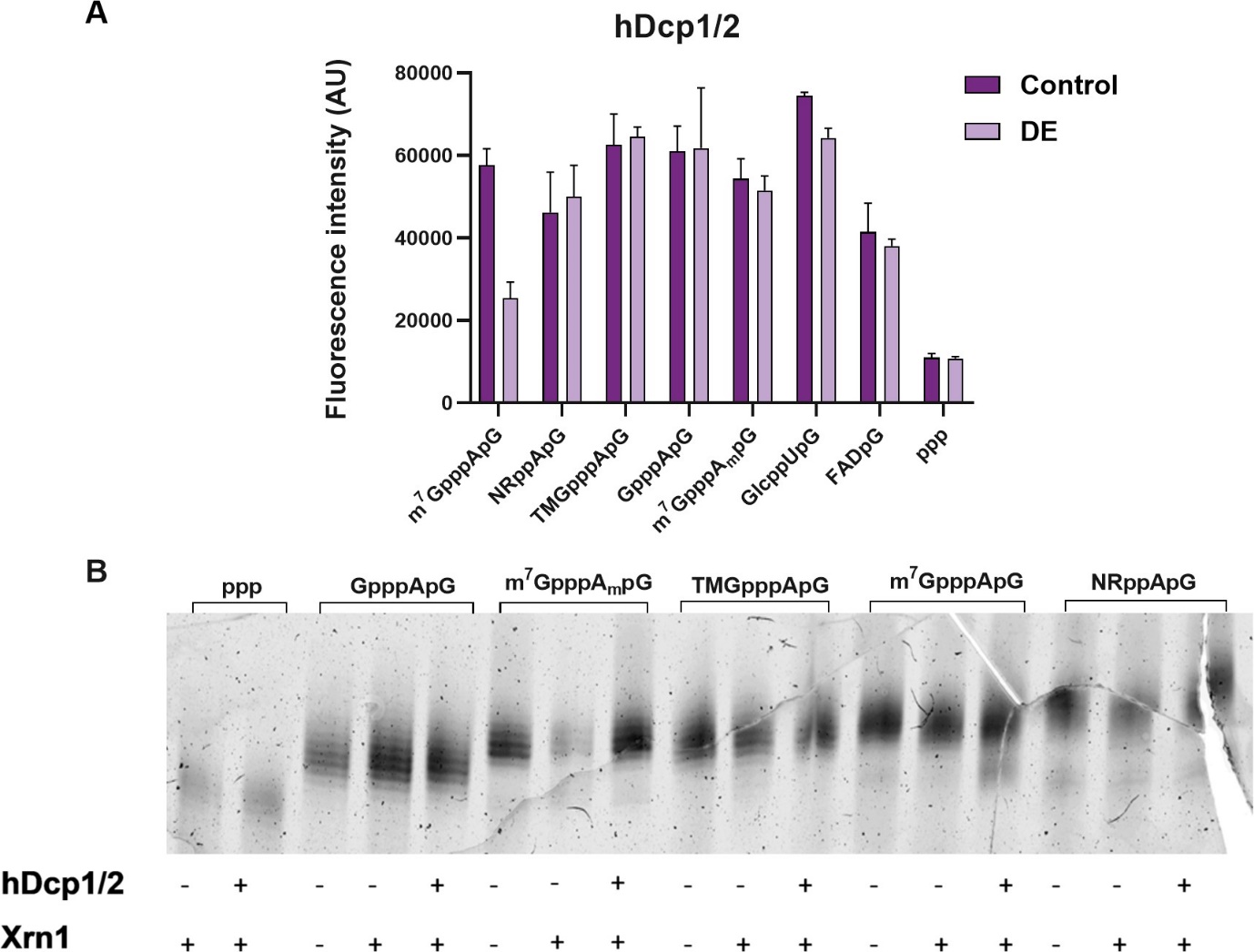


**Figure S6.** (**A**) Relative fluorescence intensity graphs for all aptamer probes used in this work. **Control**: 2 µg of capped RNA probe incubated with 2U Xrn1 for 30 minutes in 37°C. **DE**: 2 µg of capped RNA probe incubated with 50 nM hDcp1/2 complex for 30 minutes in 37°C and then with 2U Xrn1 for 30 minutes in 37°C. Every bar is mean ± SD of at least 3 independent experiments. Gel images for GlcppUpG- and FADpG- RNAs are shown in Figure S12. (**B**) Validation by boronate affinity electrophoresis (APB-PAGE) of the decapping FLINT assay with 50 nM hDcp1/2 and 2 µg of co-transcriptionally capped RNA.

## Human decapping scavenger protein (hDcpS)


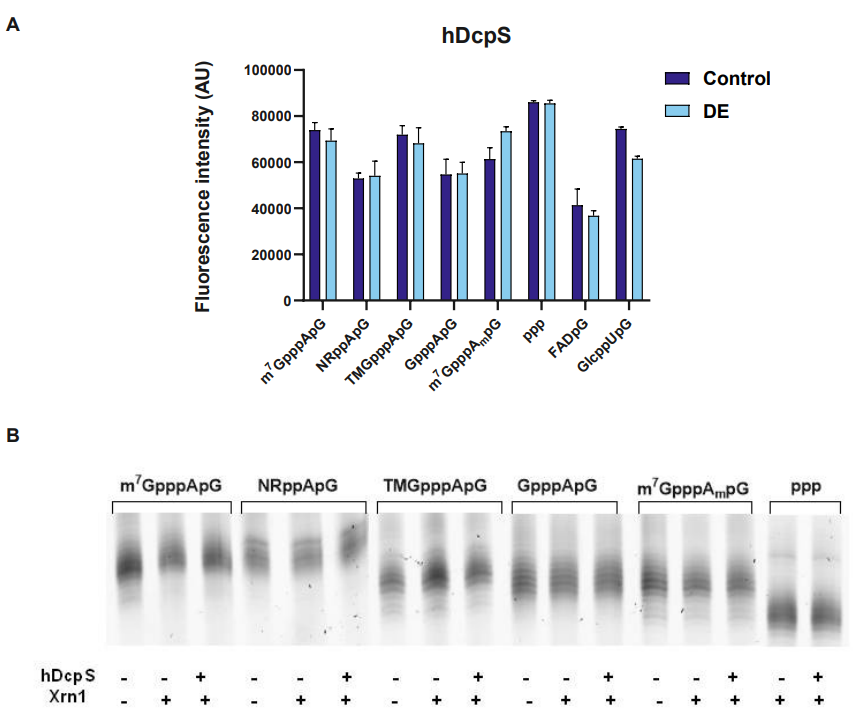


**Figure S7.** (**A**) Relative fluorescence intensity graphs for all aptamer probes used in this work. **Control**: 2 µg of capped RNA probe incubated with 2U Xrn1 for 30 minutes in 37°C. **DE**: 2 µg of capped RNA probe incubated with 50 nM hDcpS monomer for 30 minutes in 37°C and then with 2U Xrn1 for 30 minutes in 37°C. Every bar is mean ± SD of at least 3 independent experiments. Gel images for GlcppUpG- and FADpG- RNAs are shown in Figure S12. (**B**) Validation by boronate affinity electrophoresis (APB-PAGE) of the decapping FLINT assay with 50 nM hDcpS and 2 µg of co-transcriptionally capped RNA.

## Human Nudix Hydrolase 16 (hNUDT16)


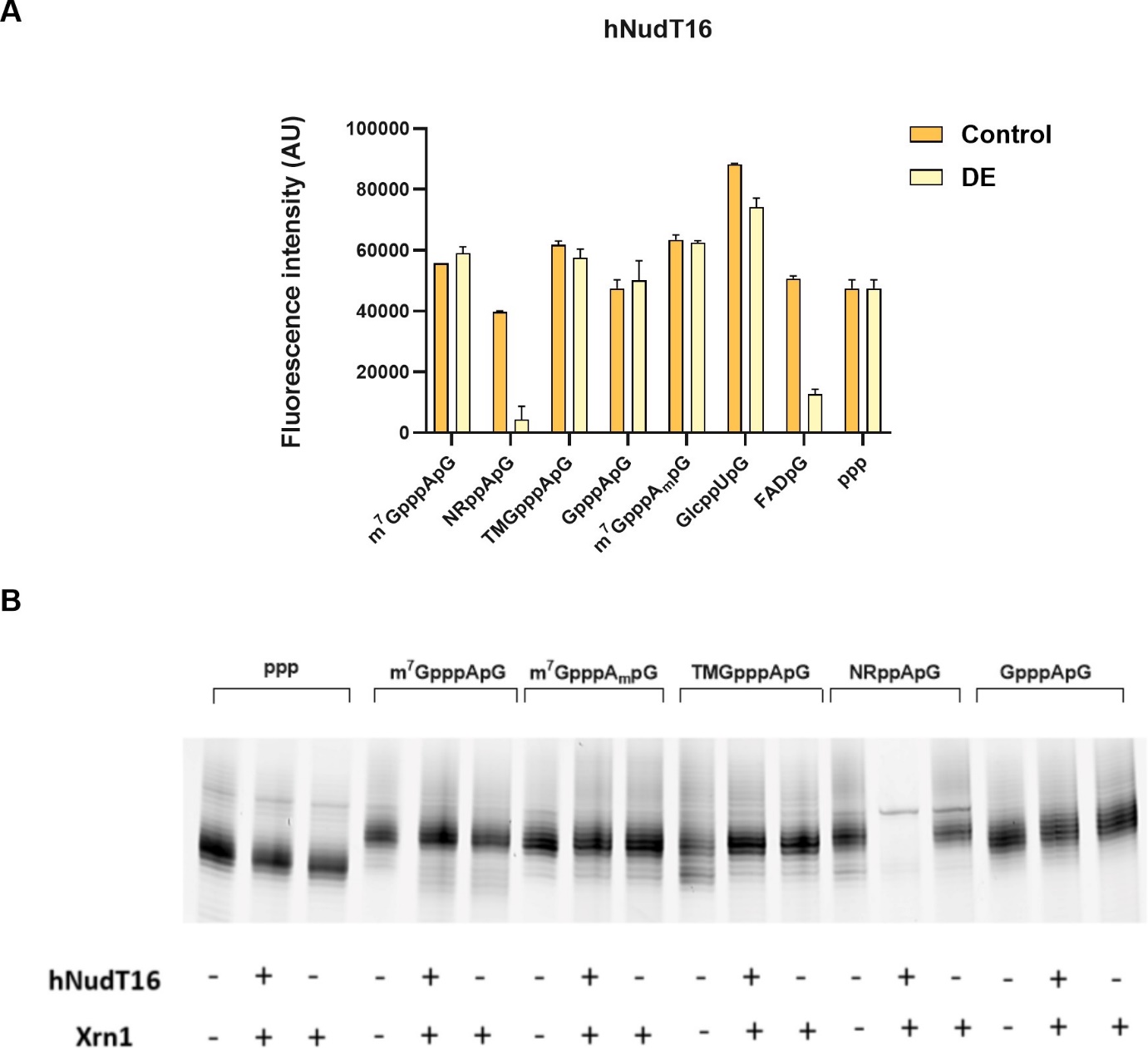


**Figure S8.** (**A**) Relative fluorescence intensity graphs for all aptamer probes used in this work. **Control**: 2 µg of capped RNA probe incubated with 2U Xrn1 for 30 minutes in 37°C. **DE**: 2 µg of capped RNA probe incubated with 50 nM hNudT16 for 30 minutes in 37°C and then with 2U Xrn1 for 30 minutes in 37°C. Every bar is mean ± SD of at least 3 independent experiments. Gel images for GlcppUpG- and FADpG- RNAs are shown in Figure S12. (**B**) Validation by boronate affinity electrophoresis (APB-PAGE) of the decapping FLINT assay with 50 nM hNudT16 and 2 µg of co-transcriptionally capped RNA.

## Vaccinia virus decapping protein D9 (VACV D9)


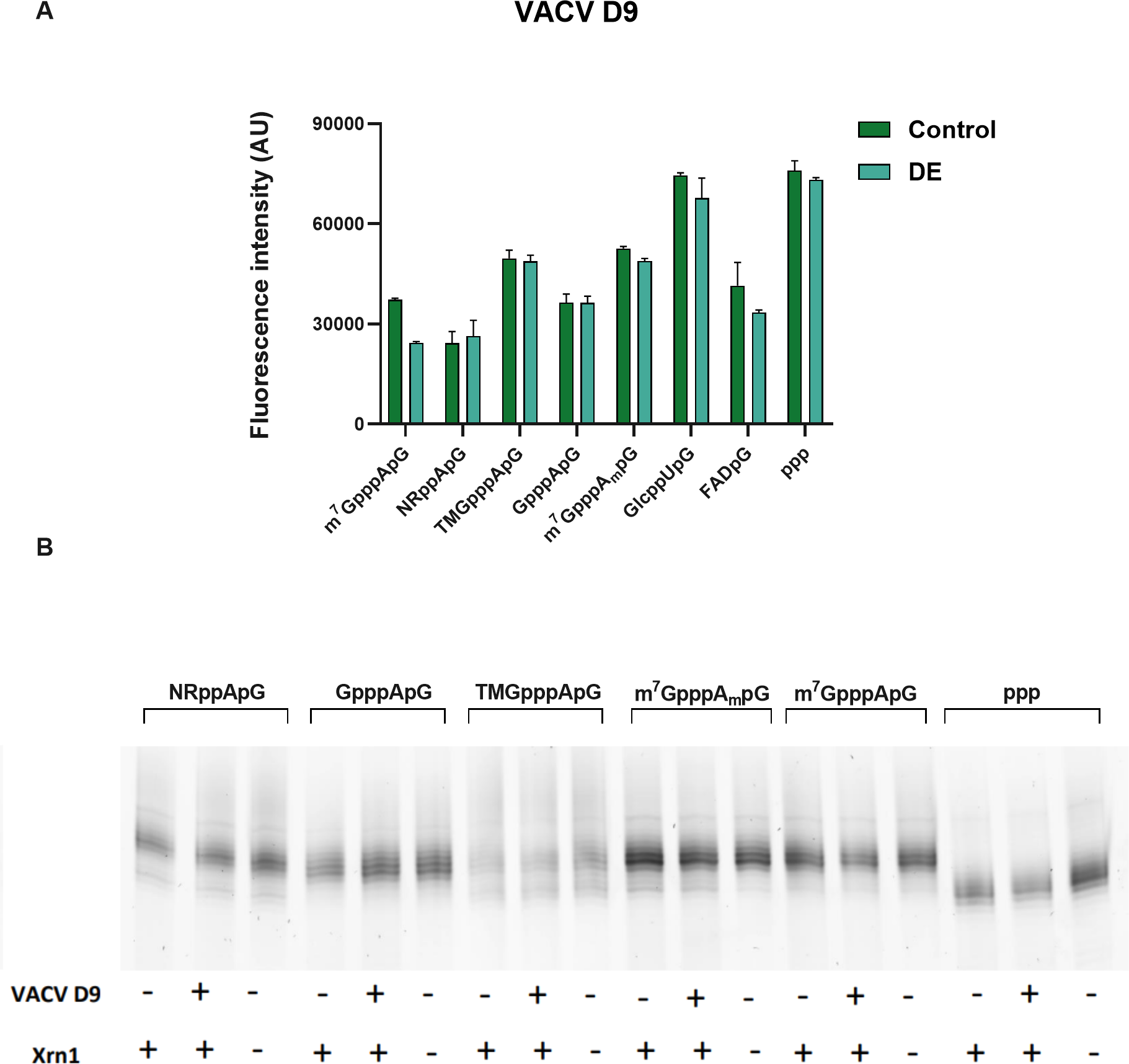


**Figure S9.** (**A**) Relative fluorescence intensity graphs for all aptamer probes used in this work. **Control**: 2 µg of capped RNA probe incubated with 2U Xrn1 for 30 minutes in 37°C. **DE**: 2 µg of capped RNA probe incubated with 50 nM VACV D9 for 30 minutes in 37°C and then with 2U Xrn1 for 30 minutes in 37°C. Every bar is mean ± SD of at least 3 independent experiments. Gel images for GlcppUpG- and FADpG- RNAs are shown in Figure S12. (**B**) Validation by boronate affinity electrophoresis (APB-PAGE) of the decapping FLINT assay with 50 nM mDXO and 2 µg of co-transcriptionally capped RNA.

## *Escherichia coli* pyrophosphohydrolase (*Ec*RppH)


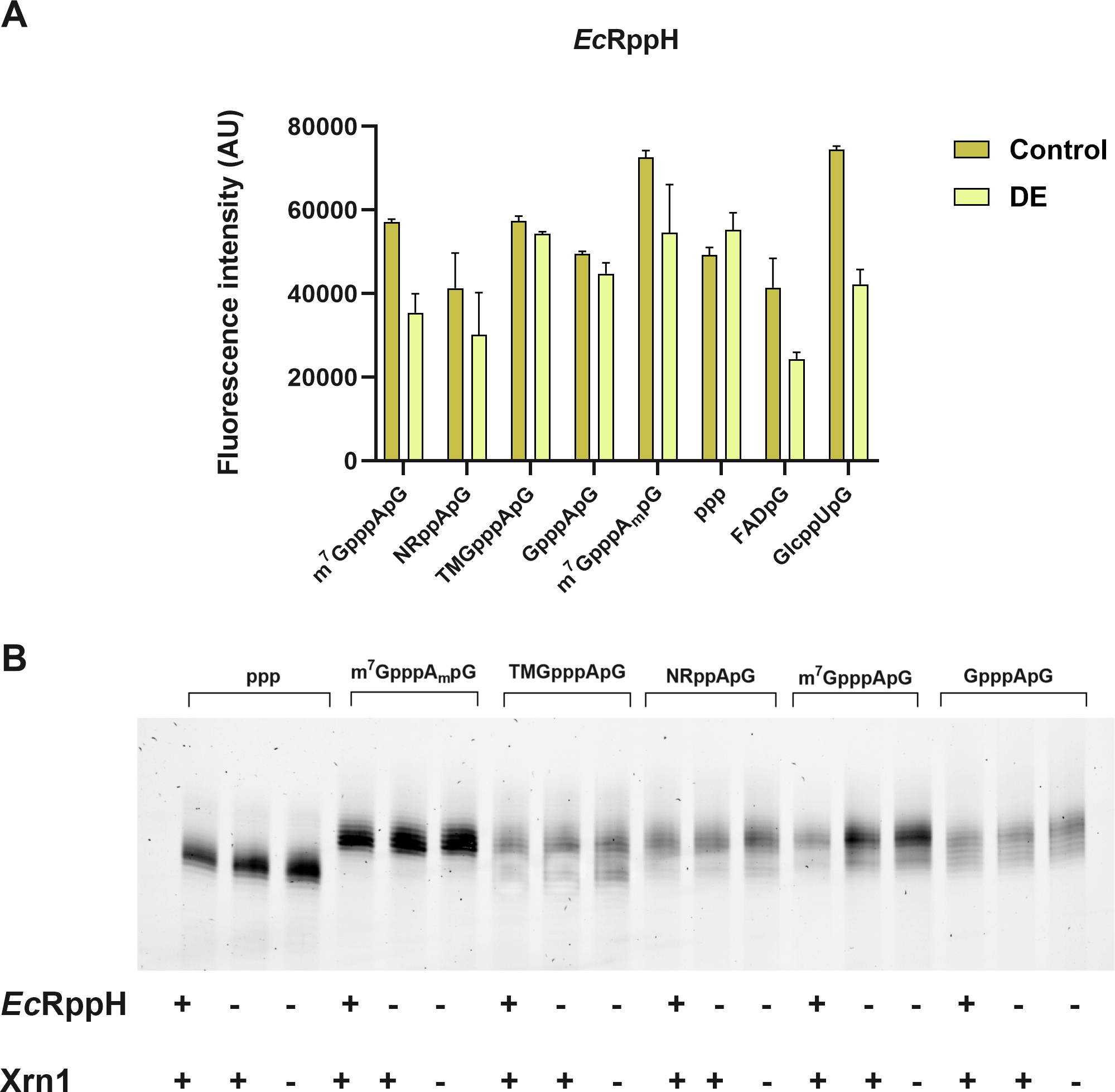


**Figure S10.** (**A**) Relative fluorescence intensity graphs for all aptamer probes used in this work. **Control**: 2 µg of capped RNA probe incubated with 2U Xrn1 for 30 minutes in 37°C. **DE**: 2 µg of capped RNA probe incubated with 50 nM *Ec*RppH for 30 minutes in 37°C and then with 2U Xrn1 for 30 minutes in 37°C. Every bar is mean ± SD of at least 3 independent experiments. Gel images for GlcppUpG- and FADpG- RNAs are shown in Figure S12. (**B**) Validation by boronate affinity electrophoresis (APB-PAGE) of the decapping FLINT assay with 50 nM *Ec*RppH and 2 µg of co-transcriptionally capped RNA.

## 5’-polyphosphatase (5’-PolyPH)


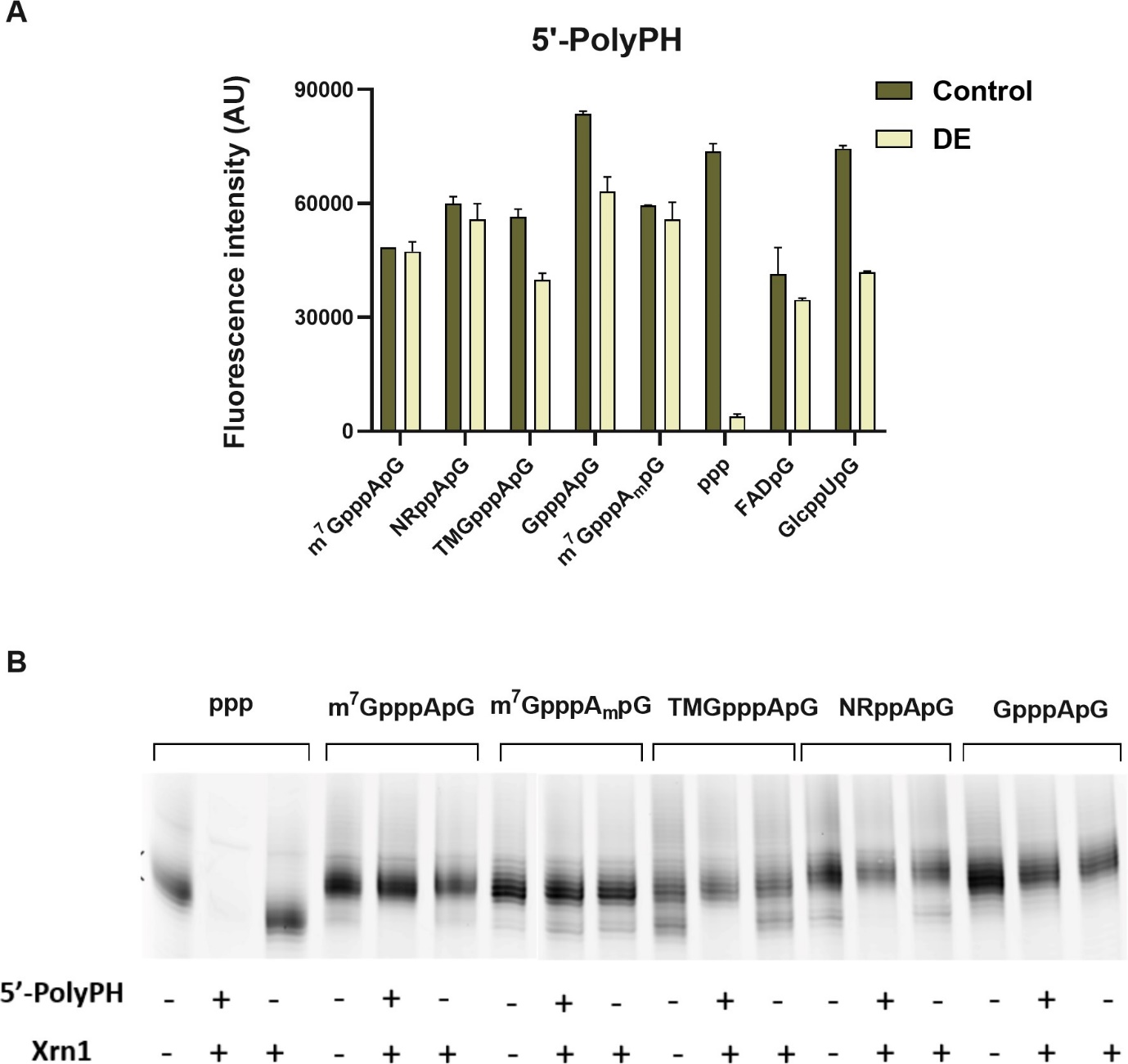


**Figure S11.** (**A**) Relative fluorescence intensity graphs for all aptamer probes used in this work. **Control**: 2 µg of capped RNA probe incubated with 2U Xrn1 for 30 minutes in 37°C. **DE**: 2 µg of capped RNA probe incubated with 50 nM 5’PolyPH for 30 minutes in 37°C and then with 2U Xrn1 for 30 minutes in 37°C. Every bar is mean ± SD of at least 3 independent experiments. Gel images for GlcppUpG- and FADpG- RNAs are shown in Figure S12. (**B**) Validation by boronate affinity electrophoresis (APB-PAGE) of the decapping FLINT assay with 50 nM mDXO and 2 µg of co-transcriptionally capped RNA.


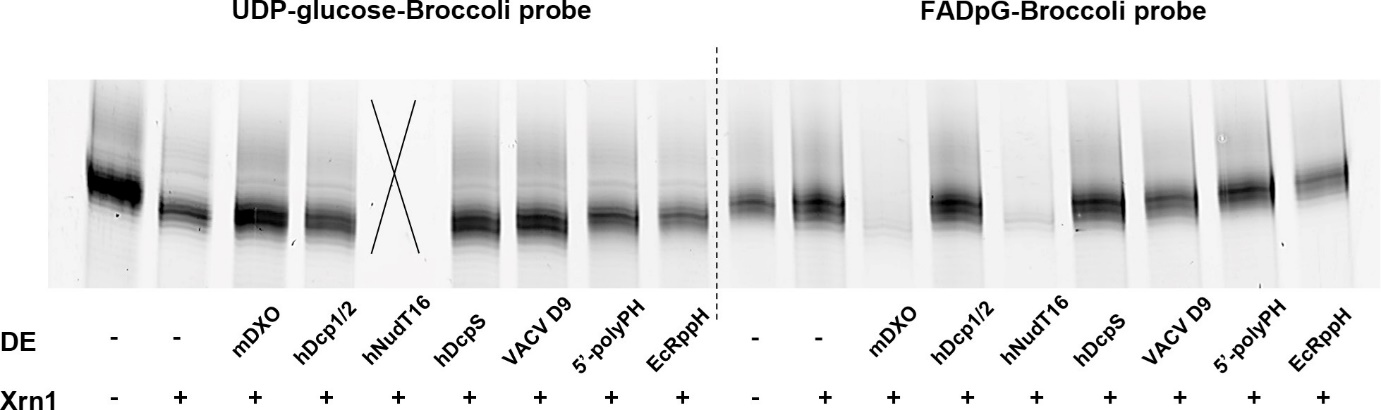


**Figure S12.** Validation by boronate affinity electrophoresis (APB-PAGE) of the decapping FLINT assay with 50 nM DE and 2 µg of co-transcriptionally capped RNA. Quantified data are shown in bar graphs in Figures S5A–S11A along with data obtained for other RNA probes.

## Evaluation of the potential for use of the FLINT assay in cell extracts


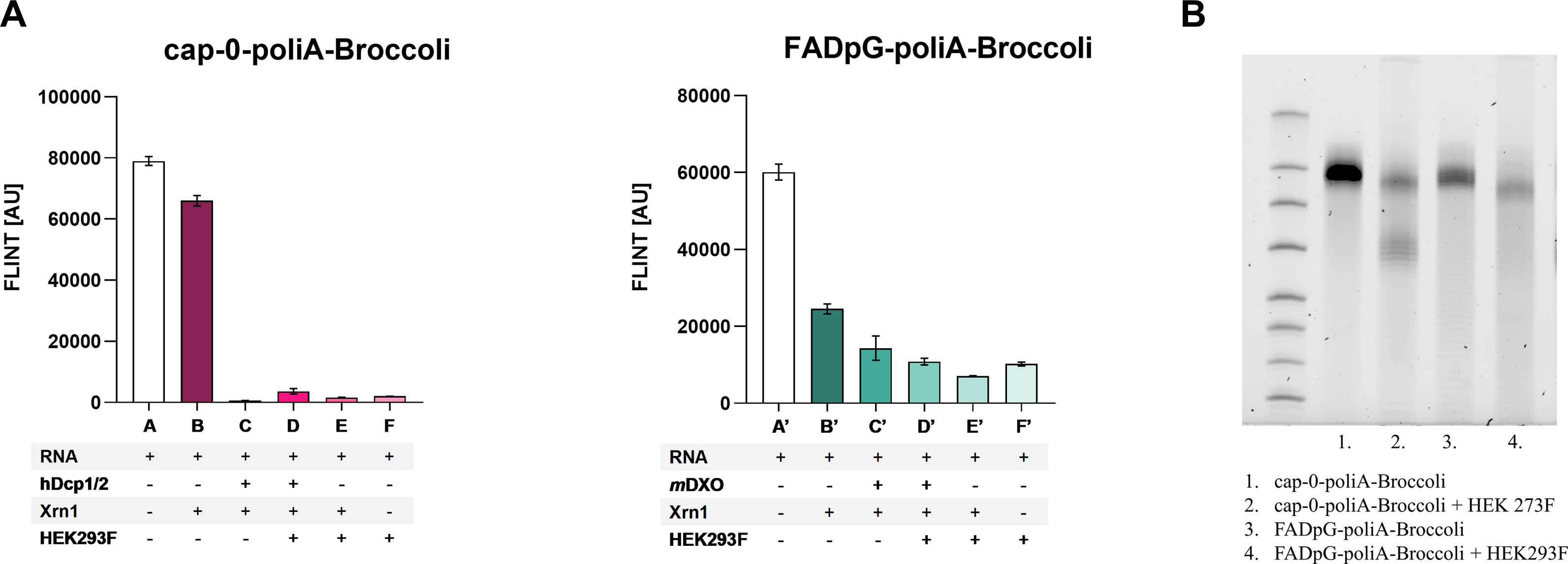


**Figure S13.** (**A**) Fluorescence intensity graphs for two aptamer probes: cap-0-RNA (**3**) and FADpG-RNA (**7**) tested in cell extracts. The exact composition of the reaction mixture is presented below the diagram. The following amounts of each compound were used for the experiment: 2 µg of capped RNA probe (**3**) or (**7**), 2U Xrn1, 1,27 µM HEK293F, 50 nM hDcp2/Dcp1 enzyme, 100 nM mDXO enzyme. Every bar is mean ± SD of at least 3 independent experiments. (**B**) Validation by gel electrophoresis (15% PAGE) of the decapping FLINT assay with 1,27 µM HEK293F and 2 µg of capped RNA probe (**3**) and (**7**).

# Chemical syntheses of cap structures

**A**

**B**

**Scheme S1.** Synthesis of (**A**) activated dinucleotide **10** (**B**) TMG cap analogue (**5**).

# Chemical structure, HRMS spectrum, NMR spectrum and HPLC chromatogram of synthesized new compound

| m_3_^2,2,7^GpppApG | |
| --- | --- |
| Chemical structure |  |
| RP HPLC | 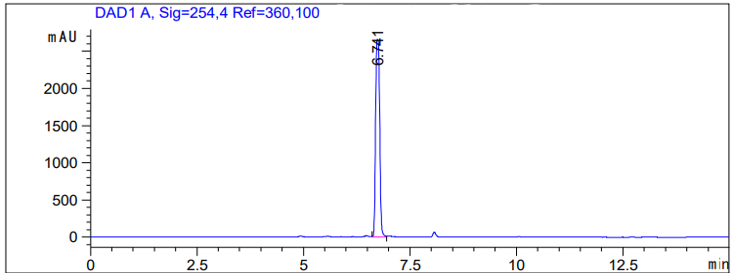 |
| HRMS (-) ESI  (Calc. [M-H]^-^ C_33_H_44_N_15_O_24_P_4_^-^ 1158.16396) | 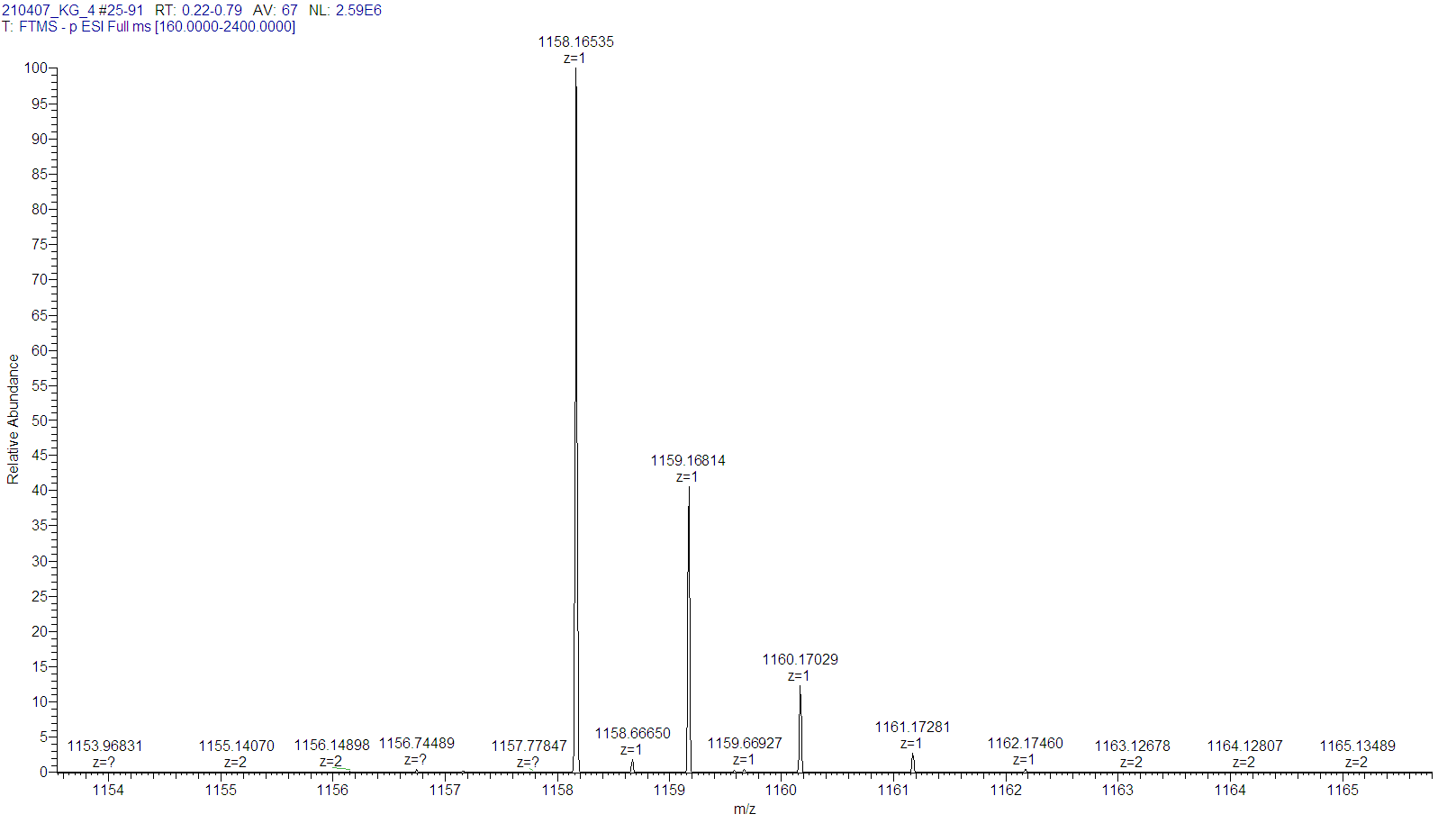 |
| ^1^H NMR (500 MHz, D_2_O, 25⁰C) |  |
| ^31^P NMR (D_2_O, 25⁰C) |  |
| COSY NMR |  |
| ^13^C HSQC NMR |  |
| ^31^P HSQC (long range) NMR |  |

# REFERENCES

1. Gruber,A.R., Lorenz,R., Bernhart,S.H., Neuböck,R. and Hofacker,I.L. (2008) The Vienna RNA websuite. *Nucleic Acids Res*, **36**, 70–74.

2. Okuda,M., Fourmy,D. and Yoshizawa,S. (2017) Use of Baby Spinach and Broccoli for imaging of structured cellular RNAs. *Nucleic Acids Res*, **45**, 1404–1415.
